# Foodborne Pathogen Prevalence and Biomarker Identification for Microbial Contamination in Mutton Meat

**DOI:** 10.3390/biology13121054

**Published:** 2024-12-16

**Authors:** Gayathri Muthusamy, Subburamu Karthikeyan, Veeranan Arun Giridhari, Ahmad R. Alhimaidi, Dananjeyan Balachandar, Aiman A. Ammari, Vaikuntavasan Paranidharan, Thirunavukkarasu Maruthamuthu

**Affiliations:** 1 Department of Agricultural Microbiology, Tamil Nadu Agricultural University, Coimbatore 641003, India; gayathri23997@gmail.com (G.M.); dbalu@tnau.ac.in (D.B.); 2 Centre for Post Harvest Technology, Agricultural Engineering College and Research Institute, Tamil Nadu Agricultural University, Coimbatore 641003, India; vag@tau.ac.in; 3 Department of Zoology, College of Science, King Saud University, Riyadh 11451, Saudi Arabia; ahimaidi@ksu.edu.sa (A.R.A.);; 4 Department of Plant Pathology, Tamil Nadu Agricultural University, Coimbatore 641003, India; agriparani@yahoo.com; 5 Department of Agronomy, Tamil Nadu Agricultural University, Coimbatore 641003, India; thirunavukkarasu.m@tnau.ac.in

**Keywords:** microbial analysis, meat metabolite, meat volatile, automated detection, food safety, source tracking

## Abstract

Microbial contamination and the prevalence of foodborne pathogens in mutton meat and during its slaughtering process were investigated through microbial source tracking and automated pathogen identification techniques. Samples from mutton meat, cutting boards, hand swabs, knives, weighing balances, and water sources were collected from four different retail sites in Coimbatore. Total plate count (TPC), yeast and mold count (YMC), coliforms, *E. coli*, *Pseudomonas aeruginosa*, *Salmonella*, and *Staphylococcus* were examined across 91 samples. The highest microbial loads were found in the mutton-washed water, mutton meat, and cutting board samples. The automated pathogen identification system identified *Staphylococcus* species as the predominant contaminant and also revealed a 57% prevalence of *Salmonella*. Further analysis of goat meat inoculated with specific pathogens showed distinct volatile and metabolite profiles, identified using gas chromatography-mass spectrometry (GC-MS). Multivariate statistical analyses, including principal component analysis (PCA), orthogonal partial least squares discriminant analysis (OPLS-DA), and sparse partial least squares discriminant analysis (sPLS-DA), identified potential biomarkers for pathogen contamination. The results highlight the significance of cross-contamination in the slaughtering process and suggest the use of volatile compounds as potential biomarkers for pathogen detection.

## Simple Summary

This study analyzed microbial contamination in mutton meat and during its slaughter process at four retail sites in Coimbatore, focusing on the total microbial load and prevalence of specific pathogens. Samples from mutton meat, cutting boards, hand swabs, knives, weighing balances, and water were collected. Mutton-washed water and mutton meat exhibited the highest microbial loads, particularly in terms of total plate count and coliforms. *E. coli* and *Staphylococcus* species were common, with automated identification revealing that most pathogens were of *Staphylococcus* origin. *Salmonella* was detected in 57% of the mutton samples using an automated identification system. Gas chromatography and mass spectrometry analysis of goat meat inoculated with pathogens identified distinct volatile and metabolite profiles, providing potential biomarkers for contamination. Multivariate statistical analysis further differentiated the volatile and metabolite profiles. These findings underscore the importance of cross-contamination during meat handling and suggest using volatile compounds for pathogen detection.

## 1. Introduction

Foods contain diverse microbes, including harmful pathogens that threaten food safety. Pathogens cause food infections through growth and illness, while food intoxications result from toxins produced by microbes like bacteria, fungi, and viruses, leading to food poisoning [1,2]. The World Health Organization estimated that approximately 2.2 million deaths occur globally each year due to diseases originating from pathogens such as *Listeria monocytogenes*, *Escherichia coli*, and *Campylobacter jejuni*. Timely detection of pathogens not only minimizes the risk of foodborne outbreaks but also enhances product assurance. Hence, developing robust, rapid, and reliable methods for detecting pathogens in various food products is essential to hamper the spread of these pathogens. India has the world’s largest buffalo population and second-largest cow and goat population. In India, the meat sector grew fast, at a compound annual growth rate of 6%. According to the Department of Animal Husbandry and Dairying, the Government of India’s livestock census data show a 5.90% decrease in the cow population from 1992 to 2019, whereas buffalo, sheep, and goat populations increased by 30.50%, 46.23%, and 29.16%, respectively [3]. Goat rearing requires very little input, as free-rangers produce leaner red meat. Other than meat, goat is also reared for its milk and leather. Despite its benefits for nutrition, employment, and income, goat meat remains undervalued in the meat trade market. The lack of structure in the goat meat sector leads to poor quality [4].

The meat-processing industry involves slaughtering animals, cutting and inspecting meat for safety, and processing it into products like sausages or lunch meats. The meat is packaged, delivered to stores, and sold, ensuring it is marketable and safe for consumers [5]. During processing, meat carcasses are primarily contaminated with pathogenic bacteria due to the leakage of fecal matter during major processing steps. Cross-contamination has also been identified as a major risk factor during processing. In recent years, increased demand for antibiotic-free, natural products has pushed consumers towards the organic food market [6]. This implies the necessity to map the potential routes of transmission of foodborne pathogens into poultry flocks and sheep and goat herds throughout the production and supply chain. *Staphylococcus aureus*, *Salmonella* Enteritidis, *Salm.* Typhimurium, *Listeria monocytogenes*, *Campylobacter jejuni* and *Campylobacter coli*, *E. coli* O157:H7, *Listeria monocytogenes*, and *Salmonella* have been reported as major foodborne pathogens in meat and meat products [7,8,9,10,11,12,13,14,15,16,17].

Culture-based detection is the gold standard for identifying foodborne pathogens, but it is time-consuming, delaying food safety measures. This method involves strain typing, selective enrichment, and confirmation, where media, environment, temperature, and incubation period affect the culture’s effectiveness [18]. Conventional methods are time-consuming, less sensitive, and labor-intensive. In contrast, immunological and molecular techniques offer greater speed and reliability, but they tend to be costly and labor-intensive [19].

Automated microbial identification systems are now commonly employed in clinical and food microbiology laboratories. These systems provide several advantages over traditional methods, such as reduced labor, minimized human error, higher sample throughput, and quicker test results. Examples of such systems include the VITEK, VIDAS (VITEK ImmunoDiagnostic Assay System) system, and PCR techniques [20]. VITEK and VIDAS are highly sensitive, with VITEK detecting a broad range of foodborne pathogens based on biochemical and metabolic profiles, allowing for faster identification of species like *Salmonella* and *Staphylococcus*. VIDAS, utilizing enzyme-linked fluorescence, enhances detection sensitivity by directly targeting pathogen-specific antigens or toxins, making it particularly effective for pathogens like *E*. *coli* and *Salmonella*. Both systems exhibit high specificity due to their ability to distinguish between closely related species through advanced biochemical, immunological, or fluorescence assays. Some other advanced techniques in the detection of foodborne pathogens include GCMS, Raman spectroscopy, and FTIR. The current study delves into microbial source tracking (MST) as a pivotal approach for identifying contamination sources and mitigating risks associated with foodborne pathogenic bacteria in meat and meat products. MST techniques include phenotypic methods like culture-based techniques, molecular methods such as polymerase chain reaction (PCR), biochemical methods like VITEK COMPACT 2, immunological methods exemplified by VIDAS, and spectroscopic methods such as GCMS. An array of advanced techniques, including phenotypic, molecular, biochemical, immunological, and spectroscopic methods, are used to identify contamination sources and mitigate risks associated with foodborne pathogens in meat and meat products. This multidisciplinary approach represents a significant step forward in enhancing food safety strategies.

## 2. Materials and Methods

### 2.1. Sample Collection

Samples such as mutton meat, cutting board swabs, hand swabs, knife swabs, weighing balance swabs, tap water, and mutton washed water were collected from four different locations: site 1 (3 retail shops), site 2 (3 retail shops), site 3 (3 retail shops), and site 4 (3 retail shops) around Coimbatore. These samples were collected and immediately analyzed for their microbial load and prevalence of foodborne pathogens.

Raw mutton meat samples: Fresh mutton samples were gathered from different butcheries in Coimbatore, Tamil Nadu, India. This study specifically targeted freshly cut mutton samples from either the forelimb or hindlimb regions. Each sample, weighing 100 g, was carefully collected under hygienic conditions in sterile polythene bags. The bags were then securely sealed and immediately placed on ice to maintain sample integrity during transport to the Food Quality Testing Laboratory, Centre for Post-Harvest Technology, Coimbatore. This collection process occurred within a few hours to preserve freshness. The samples were labeled properly, and the microbiological analysis was started immediately. Three biological replicates were maintained for each sample.
Swab samples: The swabs of the butcher’s hands, cutting board, knife, and weighing balance were collected using pre-moistened sterile swabs (HiMedia, Mumbai, India) over a defined area of several square centimeters, following the method described in [21]. The swabs were then placed into 15 mL centrifuge tubes containing sterile saline buffer. The tubes with the swabs were vortexed for 1 min, serially diluted in 9 mL of sterile peptone water, vortexed, and subsequently used for analysis.
Water samples: Tap water (fresh) and used water (for mutton washing) were sampled for microbial analysis. In the case of the fresh water, the water samples were collected after allowing the water to flow for one minute, and washed water was collected after proper homogenization in sterile 500 mL containers for further analysis [22].

### 2.2. Microbial Analysis

Microbial analysis of the collected samples was performed promptly upon their arrival at the laboratory. A total of 25 g of the mutton sample was added to 225 mL of buffered peptone water and homogenized using a stomacher. The swab samples were thoroughly homogenized using a vortex mixer. All of the samples were subjected to serial dilution and plated for further microbial analysis after homogenization.

Total plate count: The total plate count was conducted according to ISO 4833-1 (2013) [23]. For each sample, 1 mL of the diluted sample was inoculated onto plate count agar (Cat #M091, HiMedia, India) using the pour plate technique. The plates were incubated at 30 °C for 24–48 h, and bacterial colonies were counted and reported as log cfu/g (or per mL/cm^2^).
Yeasts and molds count: To enumerate yeasts and molds, 1 mL of appropriately diluted samples was inoculated onto yeast glucose chloramphenicol agar using the spread plate technique (Cat #M1590, HiMedia, India) and incubated at 25 °C for 3–5 days. The plates were examined for white, slimy yeast colonies and mycelial mold colonies. This method followed the guidelines of IS 5403:1999 [24].
Total coliforms: The appropriately diluted sample was inoculated onto a 3M Petrifilm (Cat #6414, Loughborough, UK) for coliform enumeration. A total of 1 mL of the sample was placed at the center, and the film was gently rolled back. After incubation at 37 °C for 24–48 h, red colonies with gas bubbles were counted. The results were converted to log_10_ values.
*Escherichia coli*: The presence of *E. coli* in petrifilms inoculated with the samples was tested, confirmed by the appearance of blue colonies with entrapped gas. Suspected colonies were picked using sterile toothpicks and streaked onto eosin methylene blue agar (Cat #M317, HiMedia, India), where *E. coli* exhibited a green metallic sheen. The plates were incubated at 37 °C for 24 h, and colonies showing this characteristic sheen were confirmed through PCR amplification of the *uidA* gene, which encodes β-glucuronidase, an enzyme specific to *E. coli*. Suspected colonies were further streaked onto Luria Bertani (LB) agar. After 24 h, a single colony was picked with a sterile toothpick and smeared into a PCR tube containing 20 μL of TE buffer. PCR amplification was then performed using a thermocycler following the specified conditions (Table S2). Amplicons were visualized using agarose gel electrophoresis, as described in [25].
*Escherichia coli* O157: The confirmed *E. coli* isolates were screened for Shiga toxin-producing genes by streaking them onto O157-selective medium, specifically 4-methylumbelliferyl-β-D-glucuronide *E. coli* O157 medium (Cat #M1373, HiMedia, India). The plates were incubated at 37 °C for 24 h and examined for glucuronidase activity. Non-fluorescent green colonies lacking glucuronidase activity were suspected to be *E. coli* O157. PCR amplification was performed to detect the O157-specific allele (uidA O157) and Shiga toxin genes (*stx1*, *stx2*, *eae*). Amplicons were separated using 1.2% agarose gel electrophoresis, as described previously.
*P. aeruginosa*: For *P. aeruginosa* detection, 1 mL of appropriately diluted sample was plated on Cetrimide agar (Cat #M119, HiMedia, India) to test for pyocyanin production. The plates were incubated at 37 °C for 24–48 h. The dilution ranges were as follows: 10^−5^ to 10^−6^ for mutton meat and washed water; 10^−2^ to 10^−3^ for knife swabs, hand swabs, and tap water; 10^−4^ to 10^−5^ for cutting boards. Colonies with bluish–green pigment were subcultured for fluorescence and pyocyanin production. PCR confirmation was performed using genus- and species-specific primers, followed by 1.2% agarose gel electrophoresis.
*Salmonella*: To detect *Salmonella* species, 25 g of the sample was pre-enriched in buffered peptone water (BPW) at 37 °C for 24 h. A total of 1 mL of pre-enriched sample was inoculated to selective enrichment Rappaport Vassiliadis broth at 42 °C for 24–48 h. The enriched broth was streaked onto XLD agar (Cat #M031, HiMedia, India) and incubated at 37 °C for 24 h (ISO: 6579-1:2017 [26]). Red colonies with black centers were sub-cultured on LB agar [27]. PCR amplification (Table S1) targeting the *Salmonella* invasion gene was conducted, and amplicons were resolved via 1.2% agarose gel electrophoresis for confirmation.
*Staph. aureus*: To detect *Staph. aureus*, 0.1 mL of sample diluent was inoculated onto Baird Parker agar with egg yolk tellurite emulsion (Cat #M043 & FD046, HiMedia, India). Different diluents were used for each sample type. The plates were incubated at 30 °C for 24 h, then at 37 °C for another 24 h. Positive black colonies with halo zones were observed, followed by an opaque zone around the colonies after 48 h. Suspected colonies were confirmed via PCR amplification using toxin gene-specific primers, and amplicons were analyzed via 1.2% agarose gel electrophoresis.

### 2.3. Automated Identification of Foodborne Pathogens in Meat Using VITEK 2 Compact and VIDAS

#### 2.3.1. Identification of *Staphylococcus* in Meat Using VITEK 2 Compact (BioMerieux S.A., Marcy-l’Étoile, France)

Foodborne pathogenic bacterial isolates obtained using culture-based methods were subjected to Gram staining. A single isolated bacterial colony was suspended in 3 mL of saline in a sterile test tube. A bacterial suspension with a concentration between 0.5 and 0.63 was preferred for the experiment. Based on the diagnostic Gram stain results, the appropriate VITEK 2 card or cassette was selected. The cassette and test tube racks were placed in the system, and after being loaded into the filler area, the bacterial suspensions were automatically distributed into the cassettes. The cassette was incubated for 24 h at 37 °C, after which the results were analyzed to identify the bacteria.

#### 2.3.2. Identification of *Salmonella* in Meat Using VIDAS (BioMerieux S.A., France)

Pre-enriched samples were incubated at 35 ± 2 °C for 24 ± 2 h in buffered peptone water, after which 0.1 mL of each pre-enrichment culture was transferred to 10 mL of Rappaport-Vassiliadis (RV) broth. After 24 ± 2 h of incubation at 40 ± 2 °C, 500 µL of this broth was then added to the wells of SLM strips and subjected to confirmation of the presence of *Salmonella* in the samples.

### 2.4. Biomarker Identification Using GCMS

#### 2.4.1. Inoculant Preparation

The reference strains utilized in this study were *Escherichia coli* O157:H7 STEC strain (O157-TNAU), *Staph. aureus* (ATCC 25923), *P. aeruginosa* PAO1 (cultures obtained from TNAU repository), and *Salm.* Typhimurium (a clinical isolate obtained from SRM University, Tamilnadu, India). The bacterial cultures were streaked onto sterile nutrient agar plates and incubated for 24 ± 2 h at 37 ± 2 °C. Following that, the plates were kept at 4 °C. In this experiment, a single colony from each bacterial inoculum plate was inoculated to sterile Luria-Bertani (LB) broth and cultured for 24 ± 2 h at 37 ± 2 °C with continuous shaking.

#### 2.4.2. Spiking in the Meat Matrix

The raw goat meat was spiked using the method described in [28]. Two biological replicates of raw goat meat samples of approximately 500 g each were collected. The samples were treated with 70% ethyl alcohol and air-dried under aseptic conditions to reduce surface microbial load. All of the bacterial cultures listed above were individually introduced into a surface-sterilized goat sample (150 g) enclosed in a sterile conical flask at a concentration of approximately 6 log colony-forming units (cfu) per mL of LB broth, and three replications of each biological replicates were maintained. An uninoculated raw goat meat sample was maintained as a control, and all of the samples were incubated at 37 ± 2 °C for 2 h.

#### 2.4.3. Goat Meat Volatile Collection

Goat meat volatiles were collected using thermal desorption tubes. These thermal desorption tubes were conditioned before use and were inserted into a conical flask containing goat meat samples inoculated with foodborne pathogens. This setup was then covered with a cotton plug into which the thermal desorption tubes were inserted individually into each flask, ensuring air-tight sealing. This was designed to collect the volatiles produced by the goat meat samples, and replications were maintained. These were then incubated at 37 ± 2 °C for 2 h.

#### 2.4.4. Goat Meat Metabolite Extraction

Each bacterial inoculum was individually introduced to 50 g of raw goat meat, which was allowed to incubate for 2 h at 37 ± 2 °C. Metabolite extraction was performed according to González-Peña et al. [29], with slight modifications. A pestle and mortar were used to homogenize frozen goat meat (stored at −80 °C in liquid nitrogen) into a fine powder. The powder (500 mg) and cold methanol (1 mL) were combined for 10 s using a vortex mixer. The resulting mixture was submerged in ice for 10 min, vortexed for 10 s, and then centrifuged at 14,000 rpm for 10 min (at room temperature). The supernatant was collected in 1.5 mL centrifuge tubes individually for each replication (Eppendorf, Hamburg, Germany) and kept at −80 °C before being analyzed using GC-MS.

#### 2.4.5. Goat Meat Metabolite and Volatile Profiling

The pure methanolic extract was analyzed using GC-MS (Gas chromatograph Clarus^®^ 680 and mass spectrometer Clarus^®^ SQ8C, Shelton, CO, USA). The GC-MS analyses were performed using a capillary column (30 m × 0.25 m). The oven was heated to 40 °C for 2 min, then to 250 °C at a rate of 10 °C per minute for 2 min. The detector temperature was set to 320 °C, and the input and source lines were set to 230 °C. Helium (He) was employed as a carrier gas, with a linear flow rate of 20 cm s^−1^. The MS scanning mode was adopted, with a solvent delay ranging from 0.00 to 0.50 min. The GC method was chosen with a 20 min run duration for volatiles and a 30 min run time for metabolites. The MS detector was calibrated to 200 °C, and bioactive metabolites were identified using a mass spectrum database from the National Institute of Standards and Technology Library (NIST).

### 2.5. Statistical Analysis

Microbiological analysis data from raw mutton samples were statistically evaluated using R software (Version 4.1.1) (R Core Team, Vienna, Austria). A two-way analysis of variance in randomized block design was conducted to examine the interactions between sample types and locations regarding microbiological attributes. Differences in microbial loads across locations were statistically assessed using Tukey’s honestly significant difference test (Tukey’s test) at a significance level of *p* = 0.01. A Venn diagram of the identified metabolites and volatiles was constructed using the software JVENN tool (https://jvenn.toulouse.inrae.fr/, accessed 10 August 2024). MetaboAnalyst 6.0 (https://www.metaboanalyst.ca/, accessed on 12 August 2024) was used for heat-map, hierarchical cluster, principal component analysis (PCA), partial least squares discrimination analysis (PLS-DA), sparse partial least squares—discriminant analysis (sPLS-DA), and orthogonal partial least squares—discriminant analysis (orthoPLS-DA) analysis of the different metabolites and volatile components. For more reliable results, the experiments were conducted twice, and the mean data were used for statistical analysis.

## 3. Results

### 3.1. Microbial Source Tracking and Prevalence of Mutton Meat Samples and Their Slaughtering Process

#### 3.1.1. Total Plate Count

**Figure 1:**
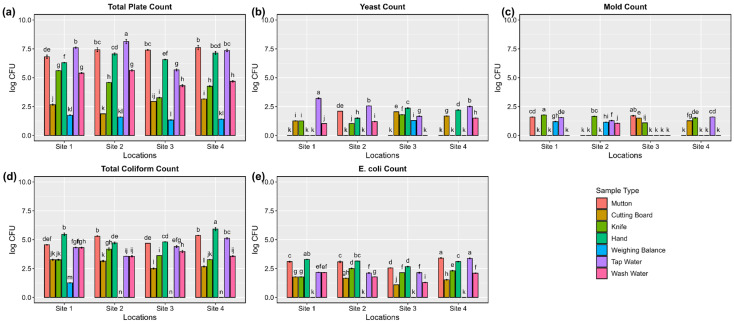
Microbial analysis of mutton and its slaughter process at varying sites. (**a**) Total plate count, (**b**) yeasts count, (**c**) molds count, (**d**) total coliform count, (**e**) *E. coli* count. Each panel represents the mean of three replicates, and the error bars indicate standard error. The panels with the same letter within a graph are not significantly different, as determined by the Tukey test (*p* < 0.01).

The TPC of mutton meat samples and slaughter process samples from varying sites are given in Figure 1 and Table S3. The microbial analysis report shows that the mutton-washed water was reported to have the highest microbial load among all the samples. Out of 91 samples, 47 samples were found to have TPC, with more than 6 log cfu/g. The highest TPC was identified for the washed water sample, which was found to have a 6-fold increase compared to the lowest TPC recorded for the tap water sample. The next highest was recorded in the mutton meat samples which had 7.14 log cfu/g. The lowest TPC was reported for the tap water, which was 1.34 log cfu/mL.

#### 3.1.2. Yeast and Mold Counts

The YMCs of mutton meat samples and slaughter-associated samples from the four locations are given in Figure 1 and Tables S4 and S5. Microbial analysis of the slaughter hygiene samples shows that the YMCs were highest in the mutton washed water samples. The lowest YMC was reported in the tap water samples, found to have a log cfu/mL value below the detection limit. The highest YMC was 3.21 cfu/mL for the mutton washed water from site 1 (Figure 1). Out of 91 samples, 30 samples were found to have more than 2 log cfu values of YMCs. The presence of molds was comparatively lower than that of yeast colonies.

#### 3.1.3. Total Coliform Count

The coliform load in the mutton meat samples and the slaughter-associated samples from all four sites are given in Figure 1 and Table S6. The coliform population was found to be more prevalent in the mutton meat samples, cutting board swabs, and mutton-washed water samples. The highest coliform count was reported in the mutton meat sample from site 4, having nearly 6 log cfu/g, followed by the cutting board (5.3 log cfu/cm^2^) and mutton-washed water (5.1 log cfu/cm^2^). The lowest coliform population was reported in the tap water which was below the detection limit. Out of 91 samples, 76 samples were found to have more than 3 log cfu/(cm^2^, mL, or g).

#### 3.1.4. *E. coli* Count

The population of *E*. *coli* was enumerated from the mutton meat samples and the samples associated with the slaughter process, and the observed log cfu values are presented in Figure 1 and Table S7. The *E*. *coli* load was found to be highest in the mutton-washed samples (3.38 cfu/mL), followed by the cutting board samples (3.34 cfu/cm^2^) and mutton meat samples (3.29 log cfu/g). The *E*. *coli* load was found to be higher in the samples from site 4. Out of 91 samples, approximately 30 samples were found to have more than 3 log cfu/(cm^2^ or ml or g). The tap water samples had the lowest *E*. *coli* count among all the sample values, being under the detection limit.

#### 3.1.5. *P. aeruginosa*

The presence of *Pseudomonas* was observed in the mutton meat, cutting board, tap water, and mutton-washed water samples. Its prevalence was higher in the mutton meat samples and mutton-washed water samples (Table 1).

#### 3.1.6. *Salmonella* sp.

The occurrence of *Salmonella* was observed in the mutton meat, cutting board, and mutton-washed water samples. Its prevalence was observed to be higher in the mutton-washed water samples (Table 1).

#### 3.1.7. *Staphylococcus* sp.

The prevalence of *Staphylococcus* was observed to be maximum in the mutton meat, hand swabs, cutting board, and mutton-washed water samples (Table 1). The presence of more pathogens in the meat and washed water might be due to cross contamination.

#### 3.1.8. Principal Component Analysis

**Figure 2:**
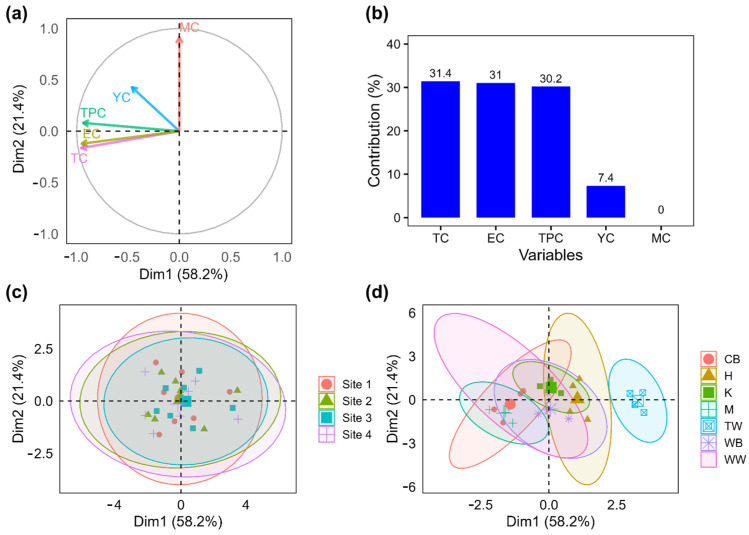
Principal component analysis relating the microbiological quality attributes to different samples. (**a**) PCA loading plot showing the orthogonal positions of the observed variables. (**b**) Percent contribution of each variable to the data divergence. PCA scoring scatter plot (**c**) grouped by sites (site 1, site 2, site 3, and site 4) and (**d**) grouped by different samples (CB—cutting board, H—hand, K—knife, M—mutton, TW—tap water, WB—weighing balance, WW—wash water).

The PCA analysis reveals that the first principal component (PC1) captures 58.2% of the total variance, and the second principal component (PC2) accounts for 21.37%. Together, these two components explain 79.56% of the dataset’s variability, highlighting their importance in differentiating sample types. The remaining components (PC3, PC4, and PC5) contribute less to the overall variance, explaining 16.27%, 3.19%, and 0.98%, respectively. This indicates that PC1 and PC2 effectively represent the data’s structure and were key for understanding sample clustering. The biplot illustrates the contributions of the TC, EC, TPC, YC, and MC variables to the first two components (Figure 2a). TC, EC, and TPC strongly influence Dim1, while YC and MC contribute less. The directions of the arrows represent correlations, with TC and EC showing a positive correlation due to their similar directions. Dim1 was primarily shaped by TC, EC, and TPC, while Dim2 reflects YC and MC. When examining the contribution of each variable to Dim1, TC, EC, and TPC each account for about 30%, while YC contributes 7.4%, and MC’s influence is minimal (Figure 2b). This suggests that the differences between the sites were largely driven by variations in TC, EC, and TPC. The PCA plots show distinct clustering patterns based on location and sample type (Figure 2c,d). Location-based clustering was more dispersed, indicating larger differences between locations, while the sample-type clusters were more compact, suggesting less variation within each group. This suggests that location has a stronger impact on overall variation than sample type, though both factors influence the data.

#### 3.1.9. Correlation Analysis

**Figure 3:**
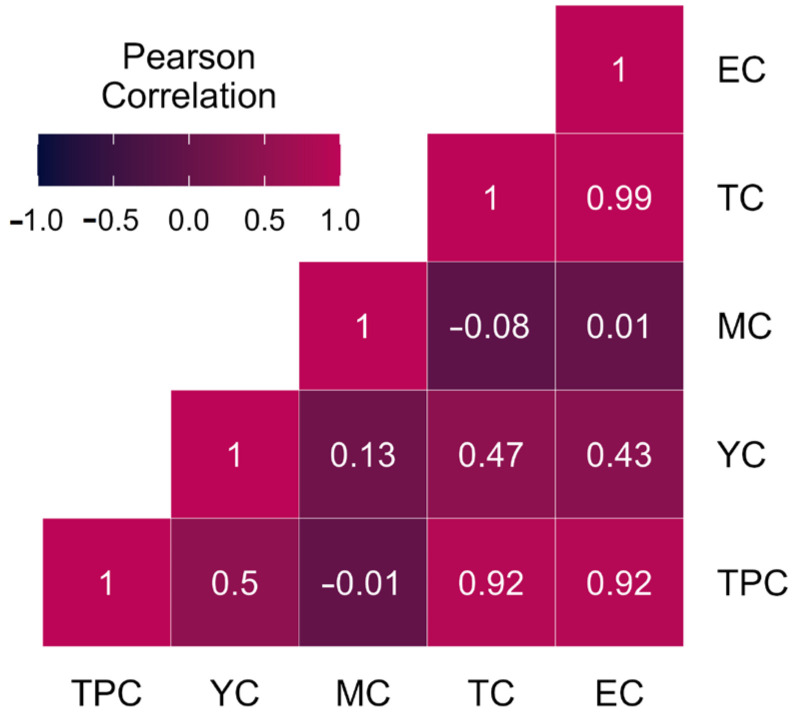
Correlation analysis of microbiological attributes of mutton meat (TPC—total plate count, YC—yeast count, MC—mold count, TC—total coliform count, EC—*E. coli* count).

The correlation matrix shows the relationships between five variables labeled TPC, YC, MC, TC, and EC. Strong positive correlations were found between TPC and EC (correlation coefficient: 1) and between TPC and TC (correlation coefficient: 0.99). A weaker negative correlation was observed between YC and MC (correlation coefficient: −0.08) (Figure 3). These results suggest that TPC and EC were highly correlated and tended to move together, while YC and MC had a weak inverse relationship. Understanding these correlations can help us to analyze the relationships between these variables and make informed decisions based on their interdependencies.

### 3.2. Outcomes of Automated Pathogen Detection in Mutton Meat Samples Using VITEK 2 Compact and VIDAS

#### 3.2.1. Automated Identification of Foodborne Pathogens Using VITEK 2 Compact for Meat Samples

Vitek 2 Compact, an automated microbial identification system, confirmed the identity of these isolates based on their biochemical profiles. The GP (Gram-positive) card was part of the Vitek 2 system, which was specifically designed for the identification of Gram-positive bacteria, including various *Staphylococcus* species. This card uses a panel of biochemical tests to rapidly and accurately identify Gram-positive organisms by assessing their metabolic reactions.

For *Staphylococcus* species, the GP card included tests such as those evaluating enzyme activities, sugar fermentation, and resistance to certain chemicals. In the secondary part, ten isolates were screened and subjected to the VITEK biochemical analysis. The organisms were identified (Table 2) using the VITEK 2 Compact system to observe the biochemical changes occurring via the incubation of the pathogens over 7 to 8 h. The obtained results showed the prevalence of *Staphylococcus*, where 7 of the 10 isolates were reported to be *Staphylococcus* sp. Most of the organisms were identified with more than 95% probability of similarity, with excellent identification.

#### 3.2.2. Automated Identification of Foodborne Pathogens Using VIDAS for Meat Samples

The VIDAS SLM Kit is based on an enzyme-linked fluorescent assay (ELFA), which enables rapid detection of *Salmonella* species in various food samples. The test result was considered positive if the relative fluorescence value (RFV) exceeded the threshold of 0.05. Samples with an RFV greater than or equal to 0.05 were classified as positive, while those with an RFV below this threshold were considered negative. This threshold value ensures reliable identification of *Salmonella* in potentially contaminated samples.

Seven mutton samples, including samples reported positive for *Salmonella* using conventional methods, i.e., site 1 (three positive): site 2 (one negative); site 3 (one negative); and site 4 (one negative and one positive), were subjected to the VIDAS SLM (*Salmonella*) Kit, which was used to test for the presence of *Salmonella*. Out of the seven samples tested, four showed positive results (site 1 and site 4) for *Salmonella*, indicating the presence of the pathogen, while the remaining 3 samples were negative. These results indicate a 57% positivity rate among the samples tested, suggesting a significant contamination level.

### 3.3. Potential Biomarker Identification Based on the Metabolites and Volatiles of Goat Meat Matrix for Detection of Foodborne Pathogens Using GCMS

Goat meat samples were individually inoculated with *Escherichia coli* O157:H7 (STEC), *Salm.* Typhimurium, *Staph. aureus*, and *P. aeruginosa* PAO1, and an uninoculated control sample was also maintained.

**Figure 4:**
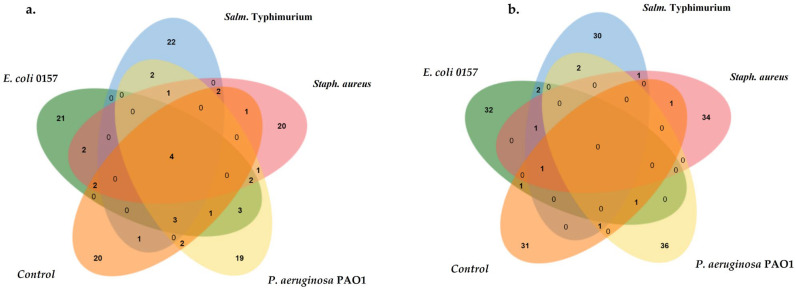
Venn diagram of metabolites (**a**) and volatiles (**b**) in control and spiked samples of goat meat.

The respective volatiles and metabolites were then identified and analyzed. Ten samples, five each for volatiles and extracted metabolites, were subjected to GC-MS analysis. Venn diagrams of the total identified metabolites (Tables S8–S12) and volatiles (Tables S13–S17) in the goat meat samples are presented in Figure 4. A total of 129 volatiles and 174 metabolites in the goat meat samples were identified. Four common volatiles in the control and pathogen-inoculated meat samples were identified, and no common metabolite was reported among the control and pathogen-inoculated samples. A total of 37 metabolites were identified as differentially expressed compounds among the identified volatiles and metabolites.

#### Analysis of Identified Volatiles and Metabolites in Control and Spiked Goat Meat Samples Based on Principle Component Analysis (PCA), Orthogonal Partial Least Squares Discriminant Analysis (OPLS-DA), and Sparse Partial Least Squares Discriminant Analysis (sPLS-DA)

**Figure 5:**
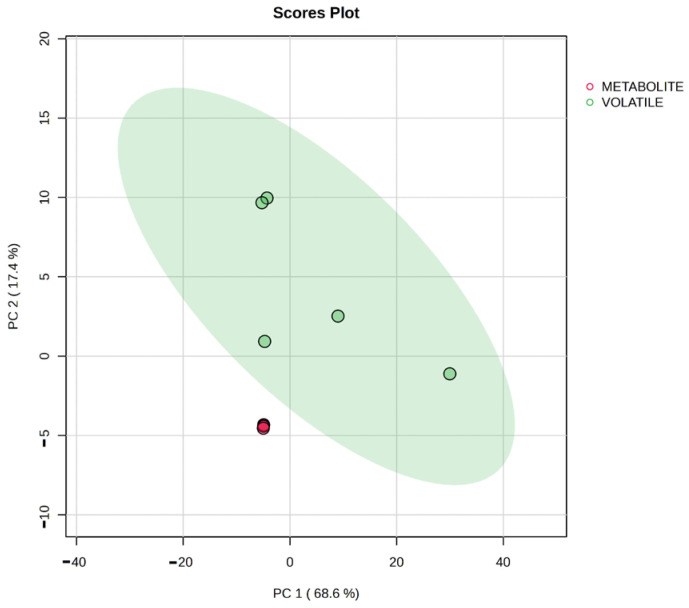
Score plot of PCA analysis of significant volatiles and metabolites in control and spiked goat meat samples.

From the obtained data, 37 compounds with significant differences among the samples were used for the PCA analysis. The PCA analysis was used to compare multivariate data that were significantly different among all the volatiles and metabolites identified. PCA score plots helped us to analyze the obtained volatile and metabolite profiles. A score plot was obtained by submitting the 37 metabolites of all 10 samples, which showed separate clusters of metabolites and volatiles (Figure S2). Principal component 1 (PC1) separated the volatiles and metabolites with a 68.6% variance. Principal component 2 (PC2) separated the volatiles and metabolites with 17.4% of the total variance. To some extent, PC2 contributed to identifying the variance across the volatiles and metabolites (Figure 5).

A new set of comprehensive variables was created by linearly recombining all of the initially identified metabolites using PCA, an unsupervised dimension reduction technique, whereas OPLS-DA is a multivariate statistical analysis technique that uses supervised pattern recognition. Figure S3 shows the correlation coefficient peak patterns of the 25 highly discriminative compounds among volatiles and metabolites in control and spiked goat meat samples, and Figure S4 presents a scatterplot of the PCA analysis highlighting significant volatiles and metabolites in these samples. The OPLS-DA analysis allowed us to identify the significantly different metabolites by the ranking of variable importance in the projection (VIP) score given in Figure S5. The range of colored boxes from red to blue on the right side indicates the comparative concentration of the corresponding metabolites and volatiles in each group. This VIP score measures the variable weight value of the OPLS-DA model variables, thereby assessing the metabolite expression patterns and their overall contributions to the model. The compounds with higher discrimination potential, i.e., VIP score > 1.35, were selected.

The score plot of the OPLS-DA analysis shows that most of the volatiles and metabolites in the goat meat samples tended to stay around the point of origin, and the volatiles were mostly found on the positive axis. All of the volatiles in control and spiked samples were clustered together, as well as the metabolites, as shown in Figure S6. sPLS-DA was applied to the same dataset as the PCA and OPLSDA to develop sensitive and specific discrimination models. The sPLS-DA analysis provided more deliberate information to identify specific metabolites that could be potential biomarkers, as it reduced the number of variables per component, thus making the model more robust and easier to interpret. In this study, the sPLS-DA analysis was performed with only 10 discriminate metabolites, as shown in the loading plot (Figure S7). The score plot of the sPLS-DA shows that component 1 has 31.8% variance, whereas component 2 has 15.1% variance among all the variables (Figure S8). Hierarchical clustering and multivariate statistical analyses, including PCA, OPLS-DA, and sPLS-DA, effectively differentiated between the volatile and metabolite profiles of the samples.

## 4. Discussion

### 4.1. Microbial Assessment of Raw Mutton Meat and Chicken Slaughter Units

The TPC results indicate that the mutton-washed water consistently had the highest microbial load across all four sampling sites, showing a 6-fold increase compared to the lowest TPC recorded in the tap water, which was slightly higher than 1 log cfu/mL. Hence, the observed microbial load in the tap water samples was not normal and could contribute to cross-contamination on surfaces such as cutting boards, knives, and weighing scales, potentially leading to an increased risk of foodborne pathogen transmission [30]. This was concerning, as a high TPC indicates a high presence of general bacteria, which could include harmful pathogens. The mutton meat also had a high TPC of 7.14 log cfu/g, reflecting potential contamination during the slaughtering process. Contaminated surfaces, such as cutting boards and knives, likely contributed to this bacterial load. Ref. [31] reported that 18% of the test mutton samples exceeded the standard microbial limits of 5 log cfu/g, which was similar to the current study. The mutton-washed water exhibited the highest YMC, with the highest value recorded at site 1. YMC were indicators of poor sanitation and spoilage. The fact that the tap water had undetectable YMC levels suggests that the water itself was not a contamination source, and cross-contamination occurred during slaughter or washing. The presence of molds was lower than yeasts, yet the detection of both indicates a potential risk of spoilage and a decreased shelf life of the meat. Ref. [32] reported that the highest *E. coli* count of 4.2 log cfu/cm^2^ was observed in the goat meat. Similarly, the presence of *E. coli* in 30 of the samples, including those taken from cutting boards and washed water, highlights poor sanitation and handling practices. The highest *E. coli* count was recorded for the cutting board swabs, suggesting cross-contamination between the meat and equipment. Ref. [33] reported the presence of *E. coli* and *Staphylococcus* in mutton. Similarly, *Pseudomonas* was more prevalent in mutton meat and washed water, known for spoiling meat and reducing its shelf life. The presence of *Salmonella* and *Staphylococcus* in washed water and meat samples was found to be the key sources contributing to cross-contamination, including improper washing, reusing unclean cutting boards, and inadequate hand hygiene. To prevent this, we recommend implementing separate water containers, sanitizing equipment after each use, and enforcing strict handwashing protocols, thereby enhancing food safety and compliance with HACCP standards.

### 4.2. Automated Detection of Foodborne Pathogens Using VITEK and VIDAS

A study in Al-Diwaniyah City [34] found *Staph. aureus* in 44% of beef burger samples from local markets, using Vitek 2 for confirmation. Conventional biochemical tests take 20 h, whereas Vitek 2 compact takes 6–8 h to deliver reports. Hence, this automated identification provides rapid and reliable reports [35]. In the present study, The Vitek 2 Compact system identified ten foodborne pathogen isolates from the mutton samples, with seven being *Staphylococcus* sp. Using biochemical profiles, it achieved over 95% similarity, confirming the high prevalence of *Staphylococcus* in the meat samples. This highlights the potential risk of harmful bacterial transmission to humans from contaminated meat. The prevalence of *Salmonella* in 400 slaughtered sheep and cattle was determined using ISO 6579 [36] and VIDAS UP methods. The results showed 3.25% and 4.25% prevalence, respectively, with dominant serovars including *Salm.* Typhimurium and *Salm.* Enteritidis, posing public health risks [37]. Similarly, in the current study, four samples out of seven were found to be positive for the presence of *Salmonella* within 48 min. While comparing the results of VIDAS with the conventional method, there was good conformity. A similar observation was made during *Salmonella* detection tests in bulk tank milk and in-line milk filters at California dairies, showing a high agreement rate of 95.75% [38].

### 4.3. Metabolite and Volatile Profiling of Raw Mutton Meat Matrix Spiked with Foodborne Pathogens for Detection of Foodborne Pathogens

Differential goat meat metabolites and volatiles in response to the individually inoculated foodborne pathogens were identified using GC-MS analysis. GC-MS is a widespread instrumental application used in food adulterant and food quality assessments [39,40]. The prevalence of foodborne pathogens such as *E. coli* O157:H7 (STEC), *Salmonella*, *Staphylococcus*, and *P. aeruginosa* in meat has been reported by several researchers [41,42]. Consequently, goat meat samples were individually inoculated with *E. coli* O157 (STEC), *Salm.* Typhimurium, *Staph. aureus*, and *P. aeruginosa* PAO1, along with an uninoculated control sample. The respective volatiles and metabolites were then identified and analyzed.

Glafenin, Propanamide, 1,4,7-Trioxa-10-azacyclododecane, and Phenazepam were the volatiles found to be common among the control and *E. coli* O157:H7, *Salm.* Typhimurium, *Staph. aureus*, and *P. aeruginosa* PAO1 spiked mutton samples. Among the volatiles identified, Glafenin has previously been reported as a flavor compound in muscle tissue, such as in Chahua chicken (*Gallus gallus domesticus*) muscle [43]. Its identification in the mutton samples, both inoculated and controlled, aligns with findings from similar studies, which suggest that Glafenin may contribute to the characteristic aroma profile of the meat. The presence of this compound across both the pathogen-inoculated and control samples reinforces its potential as a naturally occurring volatile, potentially involved in muscle biochemistry or resulting from protein breakdown during sample processing. Correlation-based network analysis was performed using Cytoscape 3.2.1 software, which provides insightful information about the networking among the identified volatiles and metabolites including the pathways involved. A correlation-based network analysis showed that the volatiles and metabolites were involved in the metabolic pathways such as arachidonic acid metabolism, biopterin metabolism, lysine metabolism, tryptophan metabolism, the urea cycle, and metabolism of arginine, proline, glutamate, aspartate, asparagine, and vitamin B9 (folate) (Figure S1).

**Figure 6:**
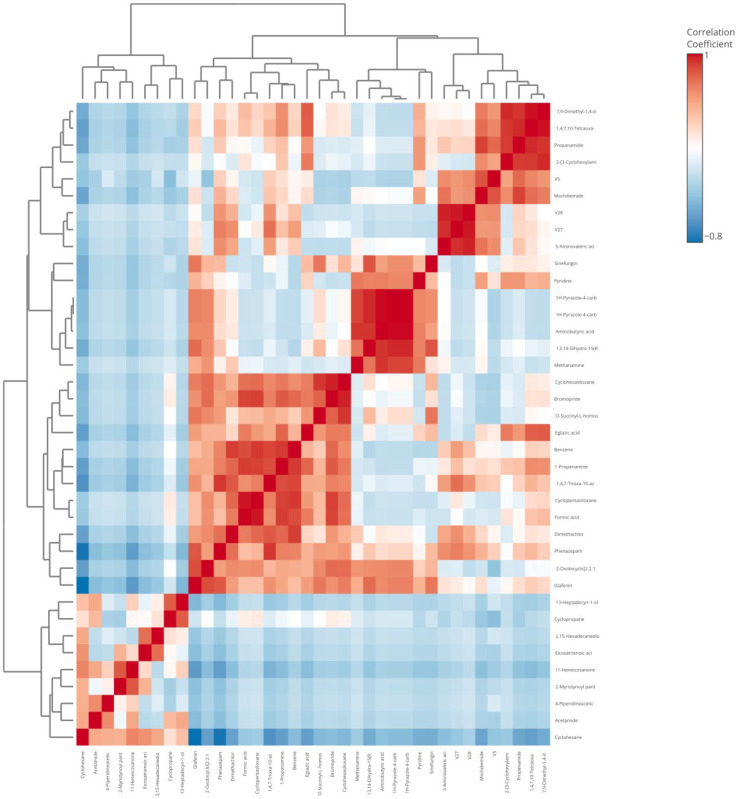
Correlation heat map visualizing the varied relationships among the volatiles and metabolites of the control and spiked goat meat samples.

To understand how biomolecules express themselves, researchers used hierarchical clustering, which entails calculating the distance matrices of data objects and then combining objects that were near to one another to form sub-clusters, helping them to understand the relationship between molecules of different samples [44]. Hierarchical clustering-based heat map visualization was obtained for the identified differential compounds using MetaboAnalyst 6.0 to observe the difference among the compounds in the control and spiked samples and also among the volatiles and metabolites identified in ten samples (Figure 6). The result showed that Propanamide, 2-[3-Cyclohexylaminopropylamino] ethylthiophosphate, 1,4,7,10-Tetraoxa-13-azacyclopentadecane was found to be higher in volatiles in the control compared to the other samples, and 5-Aminovaleric acid was also found to be highest in the *Salmonella*-spiked sample than in any of the other samples. Cyclopentasiloxane and formic acid were also reported to be highest in the *Pseudomonas* and in *Staphylococcus* 1H-Pyrazole-4-carbonitrile samples, and methanamine was reported to be highest among all samples. Propanamide was formed during the oxidation of odd-numbered fatty acids and the metabolism of some amino acids, which was found to be higher in the volatiles of the control sample than in the pathogen-spiked samples, which might be due to two reasons: it might be utilized by the inoculated bacteria, or the inoculated bacteria might be competing with the growth of natural microbiota, which involves in propenamide production. Methenamine was reported to be a bacteria-origin volatile compound [45]. In the case of metabolites of the goat meat sample, cyclopropane and 13-Heptadecyn-1-ol were reported to be highest in the control. Cyclopropane fatty acid, in particular, has previously been identified as a potential quality marker in the meat of commercial bovine species, distinguishing it from other meats such as pork, chicken, and rabbit. This compound’s higher concentration in the control sample compared to the pathogen-inoculated samples suggests that it may be more closely associated with the intrinsic quality of the meat rather than being affected by microbial contamination. Additionally, its association with commercial bovine meat and its reported potential as a quality marker in animals fed on silage align with findings from studies on livestock feeding practices [46]. Since silage feeding can influence the fatty acid profile of animal muscle tissues, the presence of cyclopropane fatty acid in goat meat may indicate similar dietary influences or quality characteristics related to livestock feeding practices [47]. Hence, cyclopropane was reported very rarely in the remaining samples. 13-Heptadecyn-1-ol, also found in high levels in the control sample, could be an indicator of meat composition or quality. Though less studied as a specific meat quality marker, the relative abundance of this compound in the control samples may indicate that it is naturally occurring in goat meat, possibly related to lipid metabolism in the muscle tissue. Further studies could assess if 13-Heptadecyn-1-ol consistently correlates with meat freshness or dietary practices, which could make it useful as an additional quality indicator. In *Pseudomonas*, 2,15-Hexadecanedione was the highest and 4-Piperidineacetic acid was reported to be the highest in *E*. *coli* O157:H7 compared to all the other samples. Based on the hierarchical clustering-based heat map visualization, it was clear that 2-Myristynoyl pantetheine metabolites were observed to be higher in the *Staphylococcus* sample than in the remaining samples. The study conducted by [48] to detect the freshness of chicken meat samples over different storage temperatures showed that the compound 2-Myristynoyl pantetheine increased in chicken meat samples after 30 days, as detected by an electronic nose. This shows that 2-Myristynoyl pantetheine could act as a biomarker in differentiating fresh meat from stale meat. The correlation heat map shows the positive and negative correlations among the variables of identified metabolites and volatiles of goat meat metabolites. Thiocyanic acid was identified as a distinctive metabolite associated with *Staphylococcus* in the current study. This finding aligns with the observations reported by Hameed [49], who also highlighted the specificity of thiocyanic acid to *Staphylococcus*. The unique presence of this compound in the metabolite profile suggests its potential role as a biomarker for the detection of *Staphylococcus* in contaminated meat matrices. Such results reinforce the importance of leveraging metabolite profiling in microbial source tracking to enhance pathogen-specific diagnostic approaches.

#### Analysis of Identified Volatiles and Metabolites Between Control and Spiked Samples of Goat Meat Based on Principal Component Analysis (PCA), Orthogonal Partial Least Squares Discriminant Analysis (OPLS-DA), and Sparse Partial Least Squares Discriminant Analysis (sPLS-DA)

GC-MS involved a typical separation of the compounds present in the volatiles and the metabolite extract of the control and spiked raw goat samples. Diverse compounds were identified using GC-MS with which potential biomarkers for detecting the presence of foodborne pathogens could be made possible through multivariate statistical analysis.

Principal component analysis is a flexible statistical technique that reduces cases-by-variables data table to their principal components, or key properties. Principal components are a small number of linear combinations of the initial variables that account for the greatest amount of variance across all the variables [50]. From the obtained data, 37 compounds with significant differences among the samples were used for the PCA analysis. The PCA analysis was used to compare multivariate data that were significantly different among all the volatiles and metabolites identified. PCA score plots helped to analyze the obtained volatile and metabolite profiles. The score plot obtained based on the 37 metabolites of all 10 samples showed separate clusters of metabolites and volatiles. Principle component 1 (PC1) separated volatiles and metabolites with a 68.6% variance. Principle component 2 (PC2) separated the volatiles and metabolites with 17.4% of the total variance. To some extent, PC2 contributed to identifying the variance across the volatiles and metabolites. A new set of comprehensive variables was created by linearly recombining all of the initially identified metabolites using PCA, an unsupervised dimension reduction technique, whereas OPLS-DA is a multivariate statistical analysis technique that uses supervised pattern recognition [51]. The OPLS-DA analysis allowed us to identify the significantly different metabolites by the ranking of variable importance in the projection (VIP) score. The range of colored boxes from red to blue on the right side indicates the comparative concentration of the corresponding metabolites and volatiles in each group. This VIP score measures the variable weight value of OPLS-DA model variables, thereby assessing the metabolite expression pattern and its overall contribution to the model. The compounds with higher discrimination potential, i.e., VIP score > 1 [52] were selected. The score plot of the OPLS-DA analysis shows that most of the volatiles and metabolites of the goat meat samples tended to stay around the point of origin, and the compounds of the volatiles were mostly found on the positive axis. All of the volatiles in the control and spiked samples were clustered together, and the same was found for the metabolites. sPLS-DA was applied to the same dataset as PCA and OPLSDA to develop sensitive and specific discrimination models. The sPLS-DA analysis provides more deliberate information in identifying specific metabolites that will be a potential biomarker, as it reduces the number of variables per component, thus making it more robust and easier to interpret the model. In this study, sPLS-DA analysis was performed with only 10 discriminate metabolites, as shown in the loading plot. The score plot of sPLS-DA shows that component 1 has a 31.8% variance, whereas component 2 has a 15.1% variance among the total variables. Hierarchical clustering and multivariate statistical analyses, including PCA, OPLS-DA, and sPLS-DA, effectively differentiated between the volatile and metabolite profiles of the samples. The analysis revealed that foodborne pathogens significantly alter the metabolic pathways, such as arachidonic acid metabolism and vitamin B9 metabolism, within the meat. This study underscores the importance of standardized guidelines and stringent regulatory oversight in the meat industry to ensure food safety. The identified biomarkers can serve as potential indicators for detecting foodborne pathogens, aiding in the development of rapid diagnostic tools upon further research. Enhanced regulatory frameworks and improved handling, processing, and storage practices were essential to mitigate the risk of contamination and protect public health. To translate biomarkers into diagnostic tools, we propose developing portable sensors for real-time detection, integrating them into existing safety protocols, and standardizing testing procedures in the future. These tools will enhance food safety by enabling early contamination detection, reducing response times, and minimizing foodborne outbreak risks. Overall, the findings contribute valuable insights into the impact of foodborne pathogens on meat quality and safety, emphasizing the need for continuous monitoring and improvement in meat production and consumption practices.

## 5. Conclusions

The current study highlights significant microbial contamination in mutton meat and associated slaughtering units, with the highest microbial load observed in mutton-washed water. Pathogens such as *P. aeruginosa*, *Salmonella*, and *Staphylococcus* were identified across various sites, emphasizing the potential for foodborne illnesses arising from improper slaughtering practices and inadequate hygiene. Advanced detection methods, including VITEK, VIDAS, and colony PCR, demonstrated high accuracy in pathogen identification, while GC-MS-based analysis revealed key volatile and metabolite biomarkers, such as Propanamide, 5-Aminovaleric acid, and Cyclopentasiloxane, associated with *E. coli* and *P. aeruginosa*.

Multivariate statistical analyses, including PCA and OPLS-DA, effectively differentiated the pathogen-inoculated samples from the controls, underscoring the utility of GC-MS in identifying pathogen-specific biomarkers. These findings highlight the potential for biomarker-based rapid detection systems, which could revolutionize pathogen monitoring in meat products. Integrating such advanced technologies into routine food safety protocols would significantly enhance contamination tracking and mitigation strategies.

To further reduce contamination risks, implementing strict hygiene protocols is essential. Regular cleaning and sanitization of equipment, frequent water changes, dedicated washing stations, and employee training on proper hygiene practices are critical. Real-time monitoring technologies and biomarker-based systems can complement these efforts, ultimately improving food safety standards and reducing foodborne illness risks.

## Figures and Tables

**Table 1:** Occurrence of *Salmonella*, *Staphylococcus*, and *Pseudomonas* in mutton meat and slaughter unit samples (“+” indicates the presence of pathogens if at least one shop within a site reports the detection of the pathogen; “−” indicates absence of pathogens).

Samples	*Salmonella*				*Staphylococcus*				*Pseudomonas*			
	Site 1	Site 2	Site 3	Site 4	Site 1	Site 2	Site 3	Site 4	Site 1	Site 2	Site 3	Site 4
Mutton	+	−	−	−	+	−	−	+	−	+	−	+
Cutting board swab	+	+	−	+	+	−	+	−	+	−	+	−
Hand swab	−	−	−	−	+	−	+	−	−	−	−	−
Knife swab	−	−	−	−	−	−	+	−	−	−	−	−
Weighing balance swab	−	−	−	−	+	−	−	−	−	−	−	−
Tap water	−	−	−	−	−	−	−	−	−	−	−	+
Mutton-washed water	+	−	−	+	+	−	−	+	+	−	−	+

Each sample from the four retail sites was tested independently in two separate experimental runs to ensure robustness and account for biological variability.

**Table 2:** Automated identification of foodborne pathogens isolates from mutton meat samples using VITEK 2 Compact.

Isolate	Time	Organism	Probability (%)
1	6.85	*Staph. hyicus*	89
2	5.85	*Staph. chromogenes*	95
3	2.43	*Staph. gallinarum*	99
4	4.62	*Staph. sciuri*	99
5	2.65	*Streptococcus iniae*	87
6	4.85	*Staph. xylosus*	97
7	4.13	*Staph. xylosus*	97
8	5.82	*Staph. xylosus*	97
9	4.82	*Staph. xylosus*	97
10	7.25	Unidentified	-

## Data Availability

The original contributions presented in this study are included in the article. Further inquiries can be directed to the corresponding author.

## Supplementary Materials

The following supporting information can be downloaded at: https://www.mdpi.com/article/10.3390/biology13121054/s1, Table S1. Specific primers used for molecular authentication; Table S2. PCR mixture for *Escherichia coli*, *Escherichia coli* O157, *P*. *aeruginosa*, *Salmonella,* and *Staphylococcus aureus*; PCR conditions; Tables S3–S7. Total plate count, yeast count, mold count, total coliforms, and *E. coli* count of mutton meat samples and slaughter unit samples collected from various sites; Figure S1. Correlation-based network analysis of volatiles and metabolites identified from the methanolic extract of control and spiked goat meat sample using Cytoscape software 3.2.1; Figure S2. Hierarchical cluster-based heat map of significant compounds of volatiles and metabolites of control and spiked goat meat samples created using MetaboAnalyst 6.0; Figure S3. Correlation coefficient peak patterns of highly discriminative 25 compound among volatiles and metabolites of control and spiked goat meat samples; Figure S4. Scatterplot of PCA analysis of significant volatiles and metabolites of control and spiked goat meat sample; Figure S5. Ranking of variable importance in the projection (VIP) score for significantly identifying metabolites via OPLS-DA analysis; Figure S6. Score plot of significant volatiles and metabolites of control and spiked goat meat samples via OPLS-DA analysis; Figure S7. Loading plot of significant volatiles and metabolites of control and spiked goat meat samples based on sPLS-DA analysis; Figure S8. Score plot of significant volatiles and metabolites of control and spiked goat meat samples based on sPLS-DA analysis; Tables S8–S17. List of metabolites and volatiles obtained using GCMS from mutton meant matrix spiked with pathogens. References [53,54,55,56] are cited in Supplementary Materials file.
