# Supplementary material for: Foodborne Pathogen Prevalence and Biomarker Identification for Microbial Contamination in Mutton Meat
Source: Biology (Basel). 2024 Dec 16;13(12):1054. doi: 10.3390/biology13121054 (PMC11673006; doi:10.3390/biology13121054)
Supplement: Supplementary file 1 [file biology-13-01054-s001.zip › biology-3274549-supplementary.pdf]

Table S1. Specific primers used for molecular authentication

Pathogenic microorganisms	Specific primers	Primer sequence	References
Pseudomonas aeruginosa	gyrPA (gyrB)	CCTGACCATCCGTCGCCACAAC<br>CGCAGCAGGATGCCGACGCC	Mohanapriya et al., 2024
E. coli O157:H7	*eae*<br><br>*stx1*<br><br>*stx2*	ATGCTTAGTGCTGGTTTAGG<br>GCCTTCATCATTTCGCTTTC<br><br>GTCACAGTAACAAACCGTAACA<br>TCGTTGACTACTTCTTATCTGGA<br><br>CGACCCCTCTTGAACATA<br>GATAGACATCAAGCCCTCGT	Padola et al., 2004
Salmonella Typhimurium	Invasion gene *invA*	TCGTCATTCCATTACCTACC<br>AAACGTTGAAAAACTGAGGA	Fratamico (2003)
Staphylococcus aureus	nuc	GCGATTGATGGTGATACGGTT<br>CAAGCCTTGACGAACTAAAGC	Sergeev et al., 2004

Table S2. PCR mixture for *Escherichia coli, Escherichia coli* O157, *P. aeruginosa, Salmonella* and *Staphylococcus aureus*

Component	Quantity (µl)
Colony suspension	1
Forward primer	0.5
Reverse primer	0.5
PCR master mix	5
nuclease-free sterile water	3

PCR condition

***Escherichia coli* and E coli O157**

PCR conditions: Initial denaturation at 95°C for 10 minutes; 40 cycles at 95°C for 1 minute (denaturation), 58°C for 1.5 minutes (annealing), and 72°C for 1 minute (extension), followed by a final extension at 72°C for 10 minutes.

P. aeruginosa

PCR conditions: Initial denaturation at 95°C for 10 minutes, followed by 40 cycles at 95°C for 1 minute (denaturation), 54°C for 1 minute (annealing for genus-specific primers) or 58°C for 1 minute (annealing for species-specific primers), and 72°C for 1 minute (extension), with a final extension at 72°C for 10 minutes.

Salmonella

PCR conditions: Initial denaturation at 95°C for 10 minutes, followed by 35 cycles of 95°C for 1 minute (denaturation), 57°C for 1 minute (annealing), and 72°C for 1 minute (extension), with a final extension at 72°C for 10 minutes.

Staphylococcus aureus

PCR conditions: Initial denaturation at 95°C for 10 minutes; 35 cycles of 95°C for 1 minute (denaturation), 55°C for 1 minute (annealing), and 72°C for 1 minute (extension); followed by a final extension at 72°C for 10 minutes

Table S3. Total plate count of mutton meat samples and slaughter unit samples collected from various sites

Sample Type	Site 1	Site 2	Site 3	Site 4
Cutting board (log cfu/g)	6.82±0.25^{de}	7.43±0.3^{bc}	7.4±0.09^{bc}	7.61±0.31^{b}
Hand (log cfu/cm^2)	2.67±0.1^{j}	1.87±0.02^{k}	2.94±0.02^{ij}	3.16±0.04^{i}
Knife (log cfu/cm^2)	5.61±0.04^{g}	4.59±0.02^{h}	3.27±0.07^{i}	4.26±0.1^{h}
Mutton (log cfu/cm^2)	6.31±0.05^{f}	7.06±0.17^{cd}	6.58±0.07^{ef}	7.14±0.24^{bcd}
Tap water (log cfu/mL)	1.75±0.05^{kl}	1.59±0.04^{kl}	1.34±0.05^{l}	1.42±0.04^{kl}
Wash water (log cfu/mL)	7.6±0.11^{b}	8.13±0.33^{a}	5.68±0.16^{g}	7.36±0.18^{bc}
Weighing balance (log cfu/cm^2)	5.4±0.1^{g}	5.64±0.11^{g}	4.31±0.15^{h}	4.69±0.12^{h}

Data are the mean of three replications. Data are presented as mean ± standard error (SE) for each parameter across the samples. Different letters (a, b, c, d) indicate significant differences between treatment groups as determined by Completely Randomized Design ($p < 0.05$).

Table S4. Yeast count of mutton meat samples and slaughter unit samples collected from various sites

Sample Type	Site 1	Site 2	Site 3	Site 4
Cutting board (log cfu/g)	BDL	2.1±0[de]	BDL	BDL
Hand (log cfu/cm^2)	1.26±0.04[i]	BDL	2.05±0.03[e]	1.68±0.04[g]
Knife (log cfu/cm^2)	1.26±0[i]	1.03±0.01[j]	1.8±0.03[f]	BDL
Mutton (log cfu/cm^2)	BDL	1.5±0.07[h]	2.37±0.08[c]	2.2±0.05[d]
Tap water (log cfu/mL)	BDL	BDL	1.3±0.01[i]	BDL
Wash water (log cfu/mL)	3.21±0.1[a]	2.56±0.01[b]	1.67±0.06[g]	2.51±0.07[b]
Weighing balance (log cfu/cm^2)	1.03±0.02[j]	1.21±0.05[i]	BDL	1.5±0.03[h]

Data are the mean of three replications. Data are presented as mean ± standard error (SE) for each parameter across the samples. Different letters (a, b, c, d) indicate significant differences between treatment groups as determined by Completely Randomized Design ($p < 0.05$).

Table S5. Mold count of mutton meat samples and slaughter unit samples collected from various sites

Sample Type	Site 1	Site 2	Site 3	Site 4
Cutting board (log cfu/g)	1.6±0.02^{cd}	BDL	1.71±0.07^{ab}	BDL
Hand (log cfu/cm^2)	BDL	BDL	1.5±0.02^{e}	1.27±0.01^{fg}
Knife (log cfu/cm^2)	1.78±0.02^{a}	1.66±0.02^{bc}	1.09±0^{ij}	1.53±0.06^{de}
Mutton (log cfu/cm^2)	BDL	BDL	BDL	BDL
Tap water (log cfu/mL)	1.2±0.05^{gh}	1.15±0.04^{hi}	BDL	BDL
Wash water (log cfu/mL)	1.55±0.04^{de}	1.29±0.06^{f}	BDL	1.6±0.01^{cd}
Weighing balance (log cfu/cm^2)	BDL	1.06±0.04^{j}	BDL	BDL

Data are the mean of three replications. Data are presented as mean ± standard error (SE) for each parameter across the samples. Different letters (a, b, c, d) indicate significant differences between treatment groups as determined by Completely Randomized Design ($p < 0.05$).

Table S6. Total coliform count of mutton meat samples and slaughter unit samples collected from various sites

Sample Type	Site 1	Site 2	Site 3	Site 4
Cutting board (log cfu/g)	4.56±0.05^{def}	5.31±0.06^{b}	4.69±0^{de}	5.37±0.03^{b}
Hand (log cfu/cm^2)	3.27±0.09^{jk}	3.15±0.11^{k}	2.51±0.09^{l}	2.67±0.09^{l}
Knife (log cfu/cm^2)	3.26±0.1^{jk}	4.18±0.18^{gh}	3.62±0.01^{i}	3.27±0.04^{jk}
Mutton (log cfu/cm^2)	5.46±0.22^{b}	4.73±0.15^{de}	4.81±0.03^{cd}	5.92±0.25^{a}
Tap water (log cfu/mL)	1.26±0.02^{m}	BDL	BDL	BDL
Wash water (log cfu/mL)	4.32±0.07^{fgh}	3.57±0.02^{ij}	4.4±0.17^{efg}	5.12±0.12^{bc}
Weighing balance (log cfu/cm^2)	4.31±0.1^{fgh}	3.56±0.1^{ij}	3.98±0.17^{h}	3.57±0.08^{ij}

Data are the mean of three replications. Data are presented as mean ± standard error (SE) for each parameter across the samples. Different letters (a, b, c, d) indicate significant differences between treatment groups as determined by Completely Randomized Design ($p < 0.05$).

Table S7. ***E. coli*** **count of mutton meat samples and slaughter unit samples collected from various sites**

Sample Type	Site 1	Site 2	Site 3	Site 4
Cutting board (log cfu/g)	3.1 ± 0.07^{c}	3.08 ± 0.13^{c}	2.55 ± 0.03^{d}	3.41 ± 0.05^{a}
Hand (log cfu/cm^2)	1.78 ± 0.02^{g}	1.66 ± 0.02^{gh}	1.09 ± 0^{j}	1.53 ± 0.06^{h}
Knife (log cfu/cm^2)	1.78 ± 0.04^{g}	2.51 ± 0.07^{d}	2.14 ± 0^{f}	2.31 ± 0.06^{e}
Mutton (log cfu/cm^2)	3.29 ± 0.02^{ab}	3.15 ± 0.03^{bc}	2.67 ± 0.06^{d}	3.1 ± 0.06^{c}
Tap water (log cfu/mL)	BDL	BDL	BDL	BDL
Wash water (log cfu/mL)	2.18 ± 0.04^{ef}	2.11 ± 0.09^{f}	2.14 ± 0.08^{f}	3.38 ± 0.07^{a}
Weighing balance (log cfu/cm^2)	2.16 ± 0.03^{ef}	1.78 ± 0.01^{g}	1.29 ± 0.05^{i}	2.1 ± 0.05^{f}

Data are the mean of three replications. Data are presented as mean ± standard error (SE) for each parameter across the samples. Different letters (a, b, c, d) indicate significant differences between treatment groups as determined by Completely Randomized Design ($p < 0.05$).

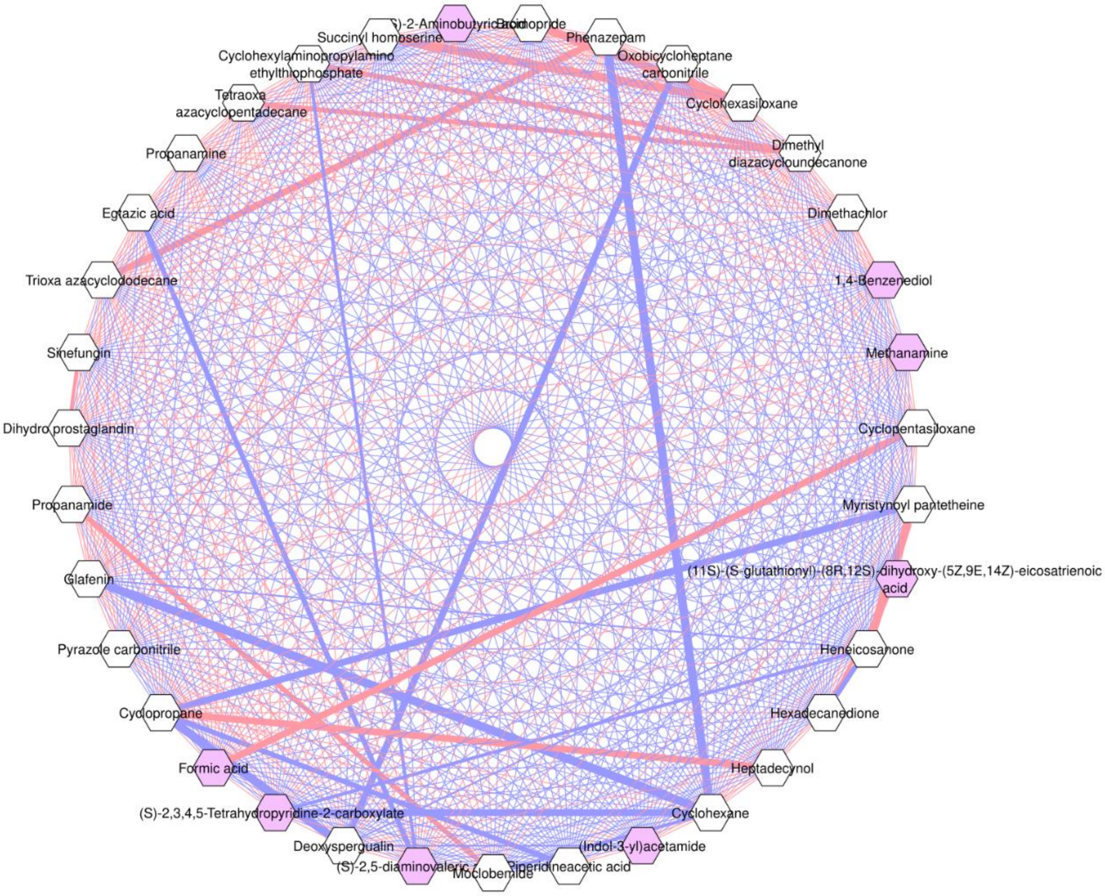

Figure S1. Correlation based network analysis of volatiles and metabolites identified from the methanolic extract of control and spiked goat meat sample using cytoscape software

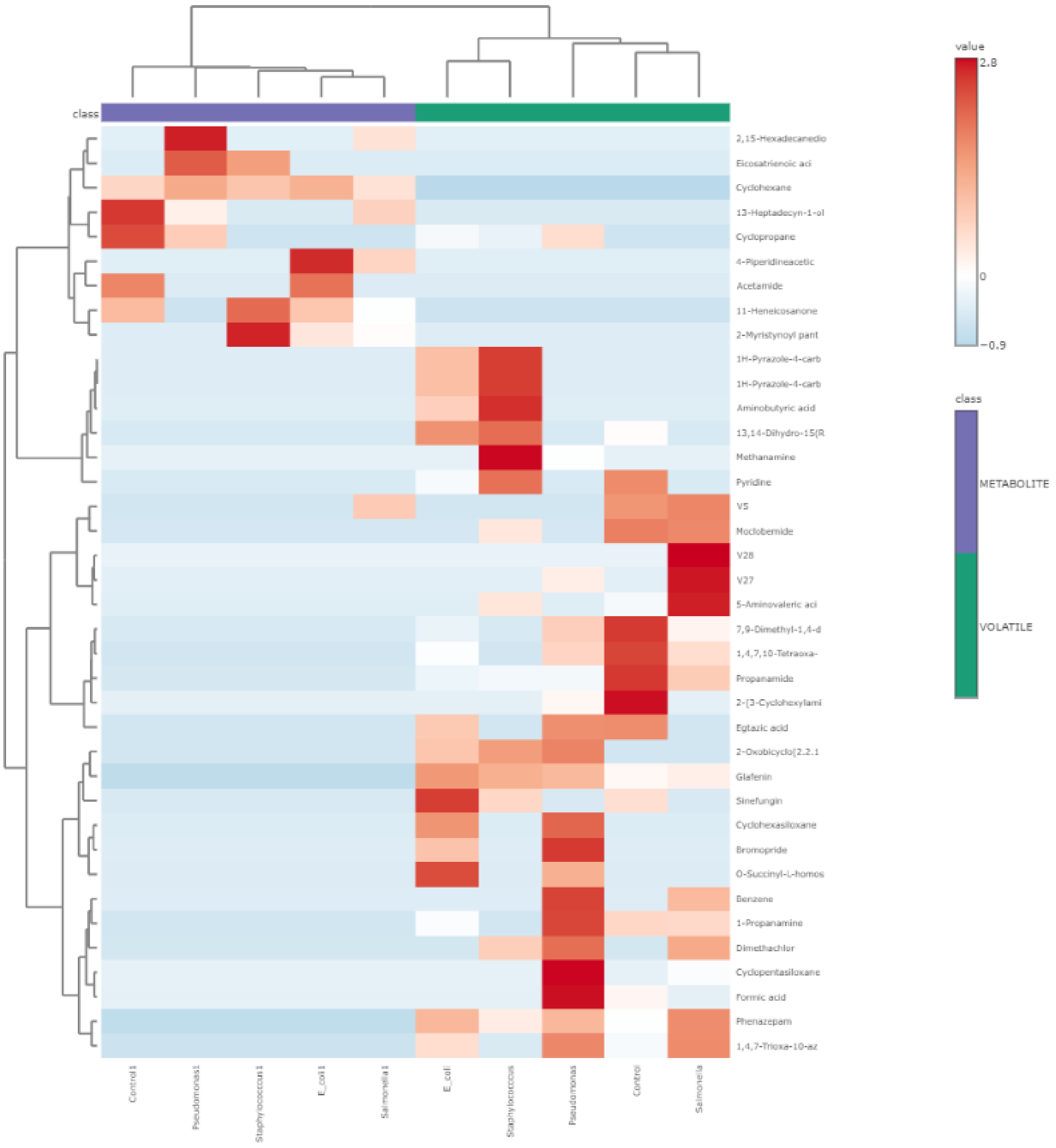

Figure S2. Hierarchical cluster based heat map of significant compounds of volatiles and metabolites of control and spiked goat meat samples done using MetaboAnalyst 6.0

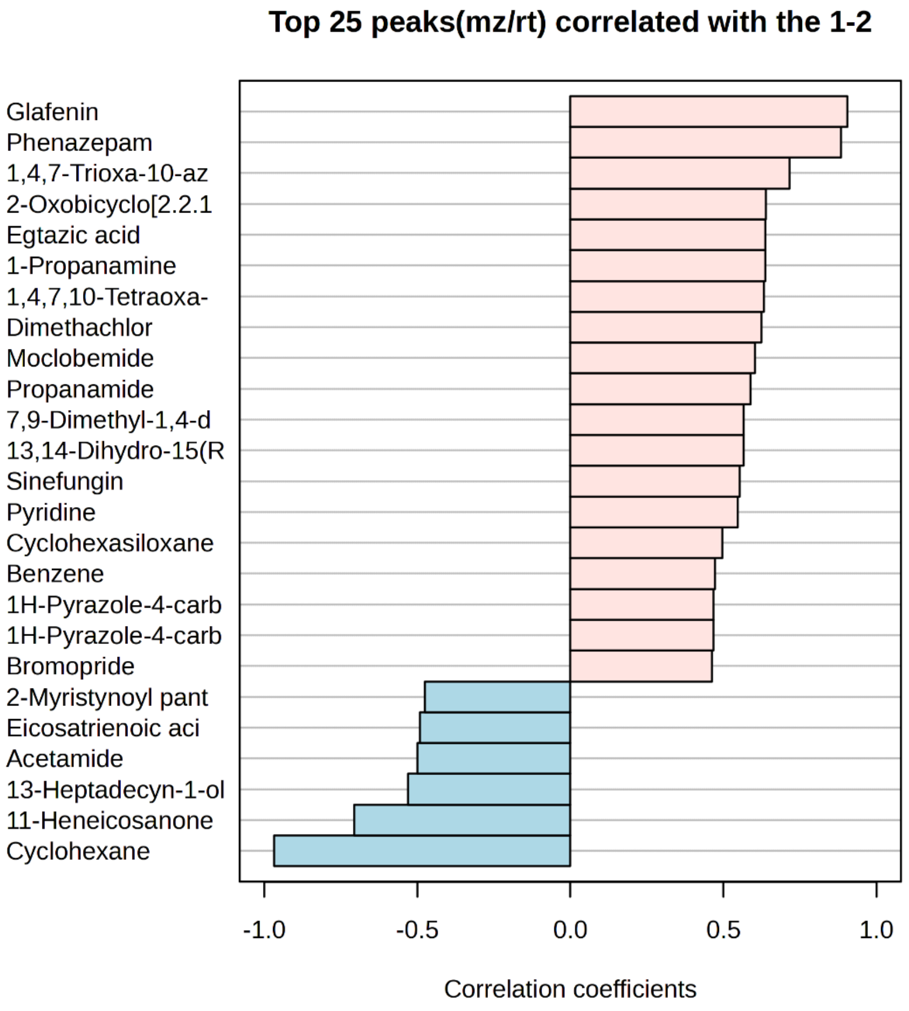

Figure S3. Correlation coefficient peak patterns of highly discriminative 25 compound among volatiles and metabolites of control and spiked goat meat sample

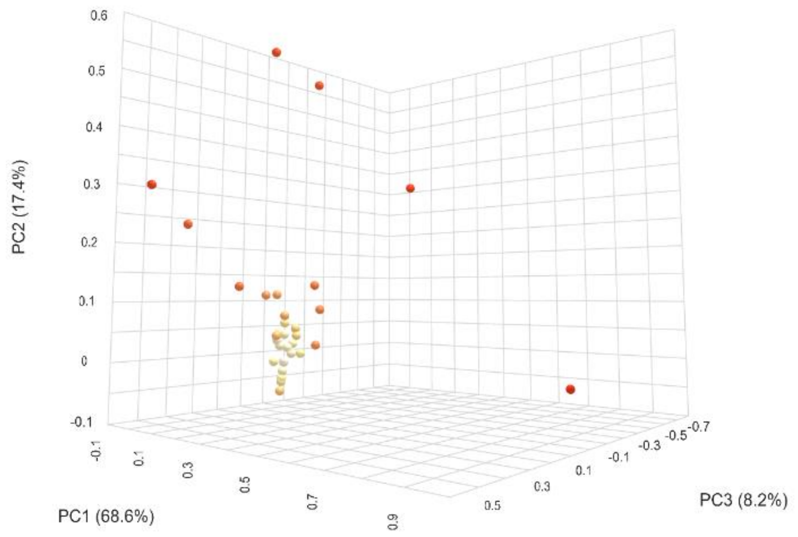

Figure S4. Scatter plot of PCA analysis of significant volatiles and metabolites of control and spiked goat meat sample

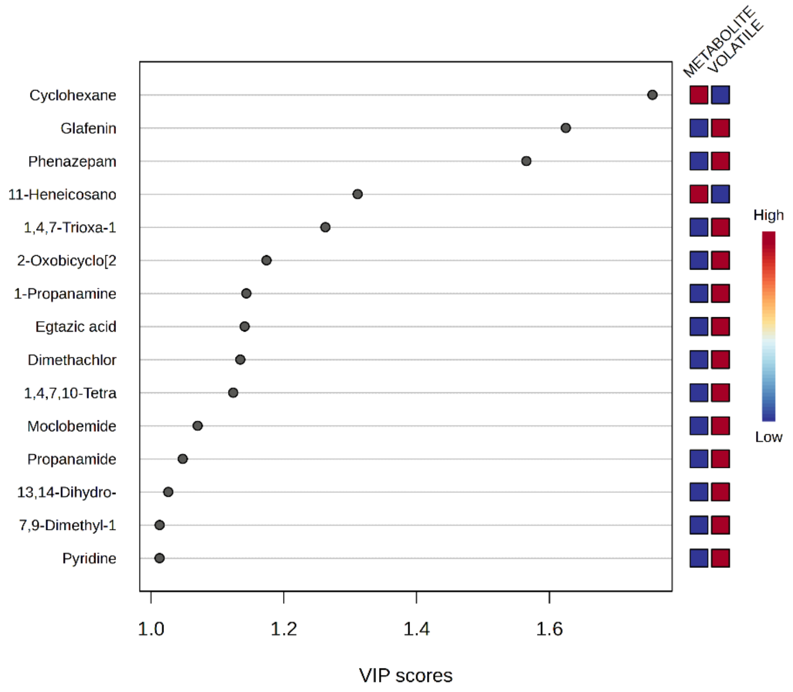

Figure S5. Ranking of variable importance in the projection (VIP) score for significantly identifying metabolites by OPLS-DA analysis

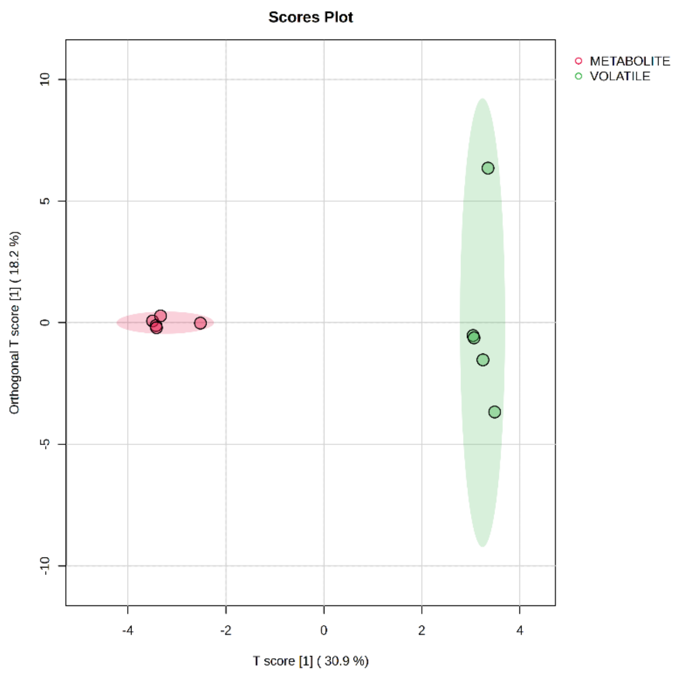

Figure S6. Score plot of significant volatiles and metabolites of control and spiked goat meat sample by OPLS-DA analysis

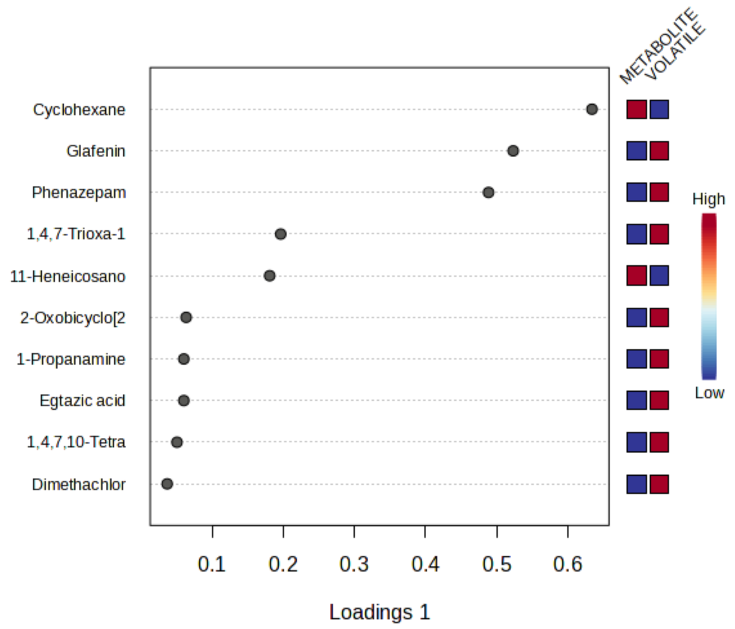

Figure S7. Loading plot of significant volatiles and metabolites of control and spiked goat meat sample sPLS-DA analysis

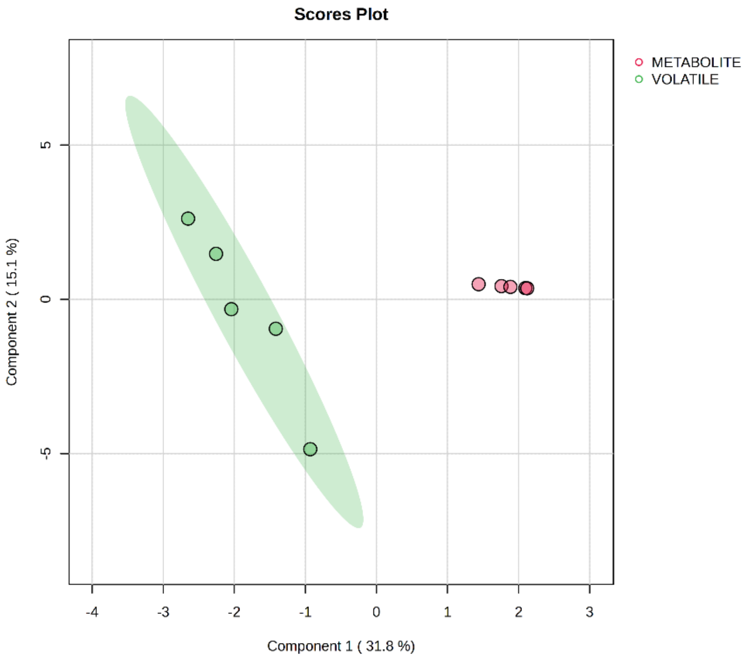

Figure S8. Score plot of significant volatiles and metabolites of control and spiked goat meat sample sPLS-DA analysis

Table S8. List of metabolites obained using GCMS from mutton meant matrix spked with *Pseudomonas*

S.NO	PEAK AREA	COMPOUND
1.	0.418	4-Cyclopentene-1,3-diol, trans-
2.	0.552	2-Hexyne
3.	0.465	Carbamic chloride, diethyl-
4.	0.449	Methyl-á-[2,4-dimethoxy-5-nitro-6-pyrimidyl]pyruvate, oxime
5.	0.47	Hitidylhistidine
6.	0.585	4-(4-Chlorobenzyl)-2-(1-methylazepan-4-yl)phthalazin-1(2H)-one
7.	0.422	13-Heptadecyn-1-ol
8.	0.58	N-Methyl-4-(2-thiophenecarboxamido)phthalimide
9.	0.427	[DTrp8,Tyr11] Somatostatin
10.	0.615	Cyclohexane, 1,1'-(1,2-dimethyl-1,2-ethanediyl)bis-, (R*,R*)-(ñ)-
11.	0.918	Cyclohexane, 1,4-dimethyl-2-octadecyl-
12.	0.721	Morphinan-4,5-epoxy-3,6-di-ol, 6-[7-nitrobenzofurazan-4-yl]amino-
13.	0.828	9,12-Hexadecadienoic acid
14.	0.443	Cyclohexane
15.	0.745	Spiro[5.5]undecane
16.	0.448	4-(1,5-Dihydroxy-2,6,6-trimethylcyclohex-2-enyl)but-3-en-2-one
17.	2.326	1-Methylcycloheptanol
18.	0.433	Amoxapine
19.	9.724	2,6,6-Trimethyl-9-undecen-1-ol
20.	0.614	3H-Cyclodeca[b]furan-2-one
21.	0.503	7-Chloro-4-[1-methyl-3-[N-methyl-N1-bromoacetylhydrazinyl]propyl]aminoquinoline
22.	0.79	8,11,14-Eicosatrienoic acid, (Z,Z,Z)-
23.	5.016	9,12-Octadecadiena
24.	0.96	5-á-Card-20(22)-enolide, 7-á,8-epoxy-3-á,11-à,14-trihydroxy-12-oxo-
25.	4.032	2,15-Hexadecanedione
26.	0.413	Dasycarpidan-1-methanol, acetate (ester)
27.	0.983	Androsterone acetate
28.	0.388	2H-Pyrrol-2-one, 4-acetyl-5-(4-chlorophenyl)-1,5-dihydro-3-hydroxy-1- [2-(1H-imidazol-4-yl)ethyl]-
29.	1.079	4,25-Secoobscurinervan-4-one, o-acetyl-16-methoxy-22-methyl-, (22à)-
30.	0.453	Furan-2-ylmethyl-(2-nitro-5-piperidin-1-yl-phenyl)-amine

Table S9. List of metabolites obained using GCMS from mutton meant matrix spked with *Staphylococcus*

S.NO	AREA	COMPOUND
1.	2.369	2-Myristynoyl pantetheine
2.	1.473	Tolazamide
3.	0.714	Fumaric acid
4.	0.615	Thiocyanic acid
5.	1.445	1,2,4,5-Tetrazine, 3,6-diethyl-
6.	0.979	propanoic acid
7.	0.739	Z,Z-2,5-Pentadecadien-1-ol
8.	0.887	7,11-Hexadecadienal
9.	0.603	7,10,13-Eicosatrienoic acid, methyl ester
10.	0.529	Oxazolidin-2-one, 4-hydroxy-3-(2-furfuryl)-4-methyl-5-spirocyclohexane
11.	0.604	9-Hexadecenoic acid
12.	0.953	Cyclopentylsilane
13.	0.634	1,1-Dimethyl-1-silacyclobutane
14.	1.014	E-1-Methoxy-3-hexene
15.	0.567	1,2-Cyclopentanediol, trans-
16.	0.598	1,2-Bis(1-methyl-3,6-diazahomoadamantantylidene-9)hydrazine
17.	0.646	D-Glucosamine, 6-sulfate
18.	0.607	Methyl 15-hydroxy-9,12-octadecadienoate
19.	0.571	2-Sulfobenzoic acid cyclic anhydride
20.	1.159	Bicyclo[2.2.1]heptane, exo-2-methyl-endo-2-acetoxy-
21.	1.016	Ethylphosphonic acid, dicyclopentyl ester
22.	1.993	Ethylphosphonic acid, dicyclopentyl ester
23.	0.702	ACTH (4-10), human
24.	0.771	Cyclohexane, 1,4-dimethyl-2-octadecyl-
25.	0.956	2-Amino-4-methoxybenzothiazole
26.	0.811	7-Chloro-3-[3,4-dichlorophenyl]-1-[[1-ethyl-3-piperidinyl]imino]-10- hydroxy-1,3,4,10-tetrahydro-9(2H)-acridinone
27.	2.28	2-Hydroxy-3-(2-thenoylmethyl)-1,4-naphthoquinone
28.	0.571	Dispiro[4.1.5.3]pentadecane-6,13-dione, 15-(3-nitrophenyl)-14-oxa-
29.	0.692	4-Acetonylcycloheptanone
30.	1.116	9,15-Octadecadienoic acid, methyl ester, (Z,Z)-
31.	1.617	11-Heneicosanone
32.	1.814	2,15-Heptadecadiene, 9-(ethoxymethyl)-
33.	1.486	Cycloundecanol, 1-methyl-
34.	0.51	5à-Androstan-16-one, cyclic ethylene mercaptole
35.	7.964	á-Sitosterol
36.	8.202	Androstan-3-one, 17-methoxy-, 3-methoxime, (5à,17á)-
37.	0.773	1,25-Dihydroxyvitamin D3, TMS derivative

Table S10. List of metabolites obained using GCMS from mutton meant matrix spked with *Salmonella*

S.NO	PEAK AREA	COMPOUND
1.	0.442	2-Myristynoyl pantetheine
2.	0.825	Tetradecanoic acid, 2-hydroxy-
3.	1.679	2-Hexyne
4.	0.696	4-Aminobutyramide,N-methyl-N-[4-(1-pyrrolidinyl)-2-butynyl]-N'-acetyl-
5.	0.398	Ethyl 5-(([3-cyano-4-(methoxymethyl)-6-methylpyridin-2-yl]sulfanyl)methyl)-1,2-oxazole-3-carboxylate
6.	0.66	Z-2-Amino-1-cyclohexanecarboxamide
7.	0.659	Tricyclo[6.3.0.0(2,6)]undecan-10-one, 3-[(2-methoxyethoxy)methoxy]- 2-methyl
8.	0.492	Heptadecane, 9-hexyl-
9.	0.548	9-Acetoxy-1-methyl-8-propyl-3,6-diazahomoadamantane
10.	0.603	Gly-Va
11.	0.584	Penta-2,4-dien-1-one, 5-dimethylamino-1-[5-(4-dimethylamino)buta-1,3- dienyl-2-thienyl]
12.	0.73	Indoline, hexahydro-5-methoxy-, cis-
13.	0.469	Benzo[c]azepin, 7,8,9-trimethoxy-
14.	0.464	Deoxyspergualin
15.	0.464	5-[(2,4-Dinitrophenyl)hydrazono]pentan-2-ol
16.	0.418	2,4-Dinitroso-1,3-naphthalenediol
17.	0.604	Cyclohexane, 1,4-dimethyl-2-octadecyl-
18.	0.456	Glucopyranuronamide
19.	0.445	11-Heneicosanone
20.	1.275	2,15-Hexadecanedione
21.	0.645	13-Heptadecyn-1-ol
22.	0.46	Octadecene
23.	0.715	1-Hexene, 2-methyl-
24.	0.393	9,19-Cyclolanostan-3-ol, 24,24-epoxymethano-, acetate
25.	0.391	3-Buten-2-one, 4-(2,6,6-trimethyl-2-cyclohexen-1-yl)-, (2,4-dinitrophenyl)hydrazone
26.	0.415	4H-Cyclopropa[5',6']benz[1',2':7,8]azuleno[5,6-b]oxiren-4-one
27.	0.812	Cyclohexane, 1,1'-dodecylidenebis[4-methyl-
28.	0.497	Pentanedioic acid, bis-dodecylamide
29.	0.602	Calconcarboxylic acid
30.	0.377	Spiro[2.3]hexane-1-carboxylic acid
31.	0.384	4-Piperidineacetic acid
32.	0.69	9-Octadecenamide, 12-hydroxy-, [R-(Z)]-
33.	0.492	Pentanedioic acid, bis-dodecylamide
34.	0.422	1-[3-(4-Amino-furazan-3-yl)-5-ethoxycarbonyl-3H-[1,2,3]triazol-4- ylmethyl]-piperidine-4-carboxylic acid ethyl ester

Table S11. List of metabolites obained using GCMS from mutton meant matrix spked with *E.coli*

S.NO	Peak area	compounds
1.	2.054	2-Propynal
2.	2.928	Butanedinitrile
3.	0.994	5-[(2,4-Dinitrophenyl)hydrazono]pentan-2-ol
4.	0.919	11,14-Eicosadienoic acid, methyl ester
5.	0.606	Folinic acid
6.	0.837	Benzene
7.	1.028	trans-Traumatic acid
8.	1.135	Mesoporphyrin IX
9.	0.656	Benzoylamide
10.	0.639	Cyclohexanol
11.	0.648	Acetamide
12.	1.341	Formamide
13.	0.84	Dihydroxanthin
14.	0.721	á-Endosulfan
15.	0.718	Behenic alcohol
16.	0.668	2-(5-Cyano-4,4,5-trimethyl-pyrrolidin-2-ylidene)-malonic acid, dimethyl ester
17.	1.159	2,8-Dioxatricyclo[5.3.0.0(3,9)]decane
18.	0.693	Carbamic acid
19.	0.692	2-Myristynoyl pantetheine
20.	0.897	8-Octadecenal
21.	0.601	Aspidofractinin-3-o
22.	0.956	N-(2-Ethylphenyl)-3-[(3-nitrobenzoyl)hydrazono]butyramide
23.	0.624	Pyrazole[4,5-b]imidazole, 1-formyl-3-ethyl-6-á-d-ribofuranosyl-
24.	0.607	8,14-Seco-3,19-epoxyandrostane-8,14-dione, 17-acetoxy-3á-methoxy4,4-dimeth
25.	0.846	Ethanone
26.	0.72	Benzeneethanamine
27.	0.808	Butanedioic acid monoamide monohydrazide
28.	1.089	N-(2-Phenylethyl)undeca-(2Z,4E)-diene-8
29.	0.877	Cyclohexane
30.	0.703	4(1H)-Pyrimidinone
31.	1.184	2-Carbomethoxy-5,5-dimethoxy quinuclidine
32.	0.706	4(1H)-Pyrimidinone
33.	0.668	Glycyl-L-histidyl-L-lysine acetate

Table S12. List of metabolites obained using GCMS from mutton meant matrix maintained as control sample

S.NO	PEAK AREA	COMPOUND
1.	0.464	6-Pentadecanol
2.	0.454	Nonenyl angelate, 2E-
3.	0.382	Deoxycytidine
4.	0.443	Acetamide
5.	0.473	Octadecanoic acid, 9,10-dichloro-, methyl ester
6.	0.602	Acetamide
7.	0.404	9,10-Secocholesta-5,7,10(19)-triene-3,25,26-triol, (3á,5Z,7E)-
8.	0.759	5-[2-Thienyl]hydantoin
9.	0.636	à-d-Xylopyranoside, methyl-2,3,4-tris-O-[9-borabicyclo[3.3.1]non-9-yl]-
10.	0.581	Aspidofractinin-3-ol, 17-methoxy-, (2à,5à)-
11.	0.412	à-d-Xylopyranoside, methyl-2,3,4-tris-O-[9-borabicyclo[3.3.1]non-9-yl]-
12.	0.498	9-Azabicyclo[4,2,1]non-2-ene, 2-acetyl-9-methyl-
13.	0.39	1H-Imidazole, 2,4,5-trimethyl-
14.	0.672	Cyclohexane
15.	3.073	2-Nonen-1-ol, 2-methyl-
16.	0.4	Ethyl iso-allocholate
17.	1.502	13-Heptadecyn-1-ol
18.	1.052	11-Heneicosanone
19.	0.451	5H-Cyclopropa[3,4]benz[1,2-e]azulen-5-one, 9-(acetyloxy)-3-[(acetyloxy)methyl]-1,1a,1b,4,4a,7a,7b,8,9,9a-decahydro-4a,7b,9atrihydroxy-1,1,6,8-tetramethyl-, [1ar-(1aà,1bá,4aá,7aà,7bà,8à,9á,9aà)]-
20.	0.4	9,10-Secocholesta-5,7,10(19)-triene-3,24,25-triol, (3á,5Z,7E)-
21.	0.831	Ursodeoxycholic acid
22.	1.47	Phorbol
23.	4.626	ç-Sitosterol
24.	4.386	Pregnan-20-one
25.	4.774	á-Sitosterol
26.	1.836	Cyclopropanebutyric acid
27.	1.434	Bonomycin hydrochloride
28.	2.101	25-Norisopropyl-9,19-cyclolanostan-22-en-24-one
29.	1.094	Cyclopenthiazide
30.	0.932	Acetic acid
31.	1.199	Pyrano[4,3-b]benzopyran-1,9-dione, 5a-methoxy-9a-methyl-3-(1-propenyl)perhydro
32.	1.552	cis-11-Eicosenoic acid
33.	0.665	Acetic acid
34.	0.871	Corynan-17-ol, 18,19-didehydro-10-methoxy-, acetate (ester)

Table S13. List of volatiles obained using GCMS from mutton meant matrix spked with *Pseudomonas*

S.NO	Peak area	compounds
1.	3.45	Oxirane
2.	2.508	1-Azabicyclo[3.1.0]hexane
3.	10.97	dl-2,6-Diaminoheptanedioic acid
4.	9.449	Formic acid
5.	7.167	Bromopride
6.	0.738	Dimethachlor
7.	0.606	3-Azetidin-1-yl-propionic acid
8.	6.966	Phenazepam
9.	0.744	Cyclopropane
10.	1.534	4-(tert-Butylamino)-6-ethylamino-5H-1,3,5-triazin-2-one
11.	0.858	O-Succinyl-L-homoserine
12.	0.391	Hydrazinecarboximidamide
13.	0.744	L-Serine
14.	0.9	Benzene
15.	0.852	9,10-Epoxy-4,8-ethanocyclohepta[c]furan-1,3-dione, hexahydro-, (3ar, 4-trans,8-trans,8a-cis,9.xi.,10.xi.)-
16.	1.37	Glafenin
17.	0.478	Bicalutamide
18.	0.888	Propanamide
19.	0.338	1-Aminocyclohexanecarboxylic acid
20.	1.018	Methanamine
21.	1.176	2-Oxobicyclo[2.2.1]heptane-1-carbonitrile
22.	0.54	Benzotriazol-1-carboxylic acid
23.	0.551	Borane
24.	0.43	Cyclopropane
25.	0.457	Cyclodecane
26.	0.408	2,2-Dimethyl-1-oxa-spiro[2.3]hexane
27.	3.678	Cyclopentasiloxane
28.	0.843	3,5-Diiodo-L-thyronine
29.	8.628	1,4,7-Trioxa-10-azacyclododecane
30.	4.963	Adenosine 2',3'-cyclic monophosphate
31.	0.458	Cyclohexasiloxane
32.	2.39	1-Propanamine
33.	1.957	7,9-Dimethyl-1,4-dioxa-7,9-diazacycloundecan-8-one
34.	0.343	Butanamide
35.	0.67	Furan
36.	0.861	1,4,7,10-Tetraoxa-13-azacyclopentadecane
37.	0.366	2-[3-Cyclohexylaminopropylamino]ethylthiophosphate
38.	0.714	Egtazic acid
39.	0.816	N-[3-[N-Aziridyl]propylidene]tetrahydrofurfurylamine

Table S14. List of volatiles obained using GCMS from mutton meant matrix spked with *Staphylococcus*

S.NO	Peak area	compounds
1.	0.364	Carnegine
2.	0.457	Ethenamine
3.	1.362	1-Penten-3-ol
4.	1.183	Moclobemide
5.	0.407	3,3-Diethyldiaziridine
6.	3.07	5-Aminovaleric acid
7.	1.796	Ethyl 2-cyanopropionate
8.	0.809	3-Acetyl-3-methyldihydrofuran-2-one
9.	2.155	L-à-Amino-ç-butyrolactone
10.	37.523	ç-Aminobutyric acid
11.	6.029	L-Alanyl-á-alanine
12.	0.419	Dimethachlor
13.	0.275	Nitenpyram
14.	0.288	3-Amino-4-methyl-pentanoic acid
15.	4.261	Phenazepam
16.	0.258	Cyclopropane
17.	1.191	1H-Pyrazole-4-carbonitrile
18.	1.244	Aziridine
19.	0.526	o-Methoxybenzonitrile
20.	0.314	D-Pyroglutamic acid
21.	0.277	Methanamine
22.	0.323	Tetracyclo[3.3.0.0(2,4).0(3,6)]oct-7-ene-4-carboxylic acid
23.	0.847	D-myo-Inositol-4-phosphate
24.	1.038	Methotrexate
25.	0.505	Pyridine
26.	1.113	2-Chloro-L-phenylalanine
27.	1.465	Glafenin
28.	0.86	Propanamide
29.	2.25	Pyridine
30.	8.233	Methanamine
31.	1.042	2-Oxobicyclo[2.2.1]heptane-1-carbonitrile
32.	5.311	13,14-Dihydro-15(R)-prostaglandin E1
33.	0.607	L-Glutamine
34.	0.719	1,4,7-Trioxa-10-azacyclododecane
35.	0.663	Sinefungin
36.	0.302	(2-Cyano-phenoxy)-acetic acid (4-hydroxy-3-nitro-benzylidene)-hydrazide
37.	0.381	1,4,7,10,13-Pentaoxa-16-azacyclooctadecane

Table S15. List of volatiles obained using GCMS from mutton meant matrix spked with *Salmonella*

S.NO	Peak area	compounds
1.	1.202	Z,Z-4,16-Octadecadien-1-ol acetate
2.	2.156	6H-1,2,5-Oxadiazolo[3,4-E]indole-6,8a-dio
3.	4.28	1-Methoxy-2-propanol
4.	2.563	Moclobemide
5.	11.135	5-Aminovaleric acid
6.	7.643	Piperidine
7.	3.88	Phenol
8.	0.713	Deoxyspergualin
9.	0.471	4,25-Secoobscurinervan-4-one
10.	10.526	Cyclopentanone
11.	4.187	Succinamide
12.	0.379	Tripropylene glycol
13.	0.562	Dimethachlor
14.	0.32	Pentapropylene glycol
15.	0.321	Guanidine
16.	0.439	Diethanolamine
17.	8.833	Phenazepam
18.	0.626	Boranamine
19.	0.447	Diethanolamine
20.	0.419	Oxetane
21.	0.522	Benzene
22.	1.852	Bicyclo[3.2.1]oct-2-ene
23.	0.84	Glafenin
24.	0.399	7,8-Dihydroneopterin
25.	2.71	Propanamide
26.	0.285	Acetyl turicine
27.	1.008	1-Propanamine
28.	0.292	Z-3,17-Octadecadien-1-ol acetate
29.	0.385	Cyclopentasiloxane
30.	8.498	1,4,7-Trioxa-10-azacyclododecane
31.	0.348	Spironolactone
32.	0.842	2,3-Di-O-methylerythramide
33.	0.283	1,1,3-Trimethylurea
34.	1.087	1,4,7-Trioxa-10-azacyclododecane
35.	0.779	1,4,7,10-Tetraoxa-13-azacyclopentadecane
36.	0.286	Octadecane
37.	1.112	7,9-Dimethyl-1,4-dioxa-7,9-diazacycloundecan-8-one

Table S16. List of volatiles obained using GCMS from mutton meant matrix spked with *E. coli*

S.NO	Peak area	compounds
1.	0.335	9-Tetradecynoic acid
2.	0.753	Butanedinitrile
3.	1.657	Uric acid
4.	1.361	Aminoguanidine
5.	4.069	Butanoic acid
6.	2.099	1H-Imidazole
7.	1.405	Tris(2-butoxyethyl) phosphate
8.	0.381	Azetidine
9.	1.336	O-Succinyl-L-homoserine
10.	16.358	ç-Aminobutyric acid
11.	11.29	Aminoacetonitrile
12.	0.85	Aminophylline
13.	3.709	Bromopride
14.	0.348	Acetaldehyde
15.	6.972	Phenazepam
16.	0.352	Cyclopropane
17.	0.651	1H-Pyrazole-4-carbonitrile
18.	0.502	Sulfanitran
19.	8.847	1-Propyne
20.	1.322	Ethanamine
21.	0.418	Pyridine-4-carboximidamide
22.	0.314	Thiocarbonohydrazide
23.	0.318	L-Cysteine
24.	1.661	Glafenin
25.	0.725	Propanamide
26.	4.363	Allyl isocyanate
27.	0.797	2-Oxobicyclo[2.2.1]heptane-1-carbonitrile
28.	0.305	Glycine
29.	0.406	2-Cyclohexen-1-one
30.	0.334	Cyclopropane
31.	4.484	13,14-Dihydro-15(R)-prostaglandin E1
32.	0.307	I-BOP
33.	4.333	1,4,7-Trioxa-10-azacyclododecane
34.	1.553	Sinefungin
35.	0.38	Cyclohexasiloxane
36.	0.505	1-Propanamine
37.	0.49	Egtazic acid
38.	0.713	1,4,7-Trioxa-10-azacyclododecane
39.	0.419	1,4,7,10-Tetraoxa-13-azacyclopentadecane

Table S17. List of volatiles obained using GCMS from mutton meant maintained as control sample

S.NO	Peak area	Compounds
1.	0.708	Ethyl isocyanide
2.	6.424	5-Aminouracil
3.	2.674	Moclobemide
4.	1.271	5-Aminovaleric acid
5.	0.665	Deoxyspergualin
6.	9.044	ç-Glu-Cys
7.	2.002	Pyridine
8.	1.805	Formic acid
9.	0.966	Tetraethylphosphonium cation
10.	3.263	Phenazepam
11.	1.132	Fluticasone propionate
12.	0.757	Glafenin
13.	6.05	Propanamide
14.	3.287	Acetic acid
15.	4.456	dl-Citrulline
16.	3.205	-(-)-Norvaline
17.	1.393	13,14-Dihydro-15(R)-prostaglandin E1
18.	0.605	Sinefungin
19.	2.063	1,4,7-Trioxa-10-azacyclododecane
20.	1.482	N-Methyl-à-aminoisobutyric acid
21.	0.72	Egtazic acid
22.	1.019	1-Propanamine
23.	4.547	7,9-Dimethyl-1,4-dioxa-7,9-diazacycloundecan-8-one
24.	1.992	1,4,7,10-Tetraoxa-13-azacyclopentadecane
25.	0.542	1-Nonene
26.	0.851	4-Nitrobenzoic acid
27.	1.11	Glycyl-L-histidyl-L-lysine acetate
28.	0.572	Dasycarpidan-8(16H)-ethanol
29.	1.086	Phenanthrene
30.	2.615	2-[3-Cyclohexylaminopropylamino]ethylthiophosphate
31.	0.556	Acetic acid, 3-acetoxy-6-(2-cyanovinyl)-3a,6-dimethyl-2,3,3a,4,5,5a,6, 9,9a,9b-decahydro-1H-cyclopenta[a]naphthalen-7-ylmethyl ester
32.	0.977	EPPS
33.	1.553	á-Amyloid / A4 Protein Precursor (APP) (328-332)
34.	1.077	Cholestan-3-one
35.	0.68	Morphinan-4,5-epoxy-3,6-di-ol
36.	0.617	2-[3-Cyclohexylaminopropylamino]ethylthiophosphate
37.	0.681	5-(3,3-Dimethyl-5-oxo-pyrrolidin-2-ylidenemethylselenyl)-2,3,3-trimethyl3,4-dihydro-2H-pyrrole-2-carbonitrile

References

Mohanapriya, Rangasamy, Vaikuntavasan Paranidharan, Subburamu Karthikeyan, and Dananjeyan Balachandar. "Surveillance and source tracking of foodborne pathogens in the vegetable production systems of India." *Food Control* 162 (2024): 110427.

Padola, N. L., Sanz, M. E., Blanco, J. E., Blanco, M., Blanco, J., Etcheverria, A.a. I., Arroyo, G. H., Usera, M. A., & Parma, A. E. (2004). Serotypes and virulence genes of bovine Shigatoxigenic Escherichia coli (STEC) isolated from a feedlot in Argentina. Veterinary Microbiology, 100, 3–9.

Sergeev, N., Volokhov, D., Chizhikov, V., & Rasooly, A. (2004). Simultaneous analysis of multiple staphylococcal enterotoxin genes by an oligonucleotide microarray assay. Journal of Clinical Microbiology, 42, 2134–2143.

Fratamico, P. M. (2003). Comparison of culture, polymerase chain reaction (PCR), TaqMan Salmonella, and Transia Card Salmonella assays for detection of Salmonella spp. in naturally-contaminated ground chicken, ground Turkey, and ground beef. Molecular and Cellular Probes, 17, 215–221.
